# Experimental colitis promotes sustained, sex-dependent, T-cell-associated neuroinflammation and parkinsonian neuropathology

**DOI:** 10.1186/s40478-021-01240-4

**Published:** 2021-08-19

**Authors:** Madelyn C. Houser, W. Michael Caudle, Jianjun Chang, George T. Kannarkat, Yuan Yang, Sean D. Kelly, Danielle Oliver, Valerie Joers, Kathleen M. Shannon, Ali Keshavarzian, Malú Gámez Tansey

**Affiliations:** 1 Department of Physiology, Emory University School of Medicine, Atlanta, GA USA; 2 Present Address: Nell Hodgson Woodruff School of Nursing, Emory University, Atlanta, GA USA; 3 Present Address: Department of Environmental Health, Emory University Rollins School of Public Health, Atlanta, GA USA; 4 Present Address: Department of Neurology, Johns Hopkins Medicine, Baltimore, MD USA; 5 Department of Neurology, University of Wisconsin School of Medicine and Public Health, Madison, WI USA; 6 Department of Internal Medicine, Rush Medical College, Chicago, IL USA; 7 Present Address: Department of Neuroscience and Neurology, Center for Translational Research in Neurodegenerative Disease, University of Florida College of Medicine, Gainesville, FL 32610 USA

**Keywords:** Parkinson’s disease, Intestine, Gut, Inflammation, Immune, Colitis, Neurodegeneration, MPTP, DSS

## Abstract

**Background:**

The etiology of sporadic Parkinson’s disease (PD) remains uncertain, but genetic, epidemiological, and physiological overlap between PD and inflammatory bowel disease suggests that gut inflammation could promote dysfunction of dopamine-producing neurons in the brain. Mechanisms behind this pathological gut-brain effect and their interactions with sex and with environmental factors are not well understood but may represent targets for therapeutic intervention.

**Methods:**

We sought to identify active inflammatory mechanisms which could potentially contribute to neuroinflammation and neurological disease in colon biopsies and peripheral blood immune cells from PD patients. Then, in mouse models, we assessed whether dextran sodium sulfate-mediated colitis could exert lingering effects on dopaminergic pathways in the brain and whether colitis increased vulnerability to a subsequent exposure to the dopaminergic neurotoxicant 1-methyl-4-phenyl-1,2,3,6-tetrahydropyridine (MPTP). We assessed the involvement of inflammatory mechanisms identified in the PD patients in colitis-related neurological dysfunction in male and female mice, utilizing mice lacking the Regulator of G-Protein Signaling 10 (RGS10)—an inhibitor of nuclear factor kappa B (NFκB)—to model enhanced NFκB activity, and mice in which CD8^+^ T-cells were depleted.

**Results:**

High levels of inflammatory markers including *CD8B* and NFκB p65 were found in colon biopsies from PD patients, and reduced levels of RGS10 were found in immune cells in the blood. Male mice that experienced colitis exhibited sustained reductions in tyrosine hydroxylase but not in dopamine as well as sustained CD8^+^ T-cell infiltration and elevated *Ifng* expression in the brain. CD8^+^ T-cell depletion prevented colitis-associated reductions in dopaminergic markers in males. In both sexes, colitis potentiated the effects of MPTP. RGS10 deficiency increased baseline intestinal inflammation, colitis severity, and neuropathology.

**Conclusions:**

This study identifies peripheral inflammatory mechanisms in PD patients and explores their potential to impact central dopaminergic pathways in mice. Our findings implicate a sex-specific interaction between gastrointestinal inflammation and neurologic vulnerability that could contribute to PD pathogenesis, and they establish the importance of CD8^+^ T-cells in this process in male mice.

**Graphical abstract:**

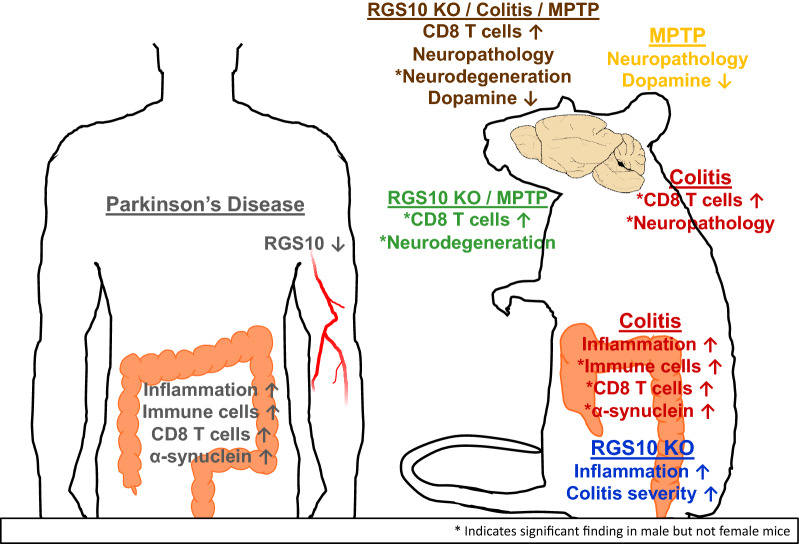

**Supplementary Information:**

The online version contains supplementary material available at 10.1186/s40478-021-01240-4.

## Background

Parkinson’s disease (PD) is the second most common neurodegenerative disorder, affecting hundreds of thousands in the United States alone [1]. Its debilitating motor impairments are caused primarily by the progressive degeneration of dopaminergic neurons in the substantia nigra pars compacta (SNpc) region of the brain. The factors that initiate neuronal dysfunction and lead to neuropathology remain unclear. It has been observed, however, that gastrointestinal (GI) problems are associated with PD and that GI symptoms commonly manifest decades before motor symptoms [2]. This finding has prompted theories that intestinal pathology could promote development of sporadic PD.

Intestinal inflammation has been proposed [3–5] as a key mechanism mediating gut-to-brain PD progression. Indicators of gut inflammation in PD include documentation of oxidative stress in colon tissue [6] and increased levels of the inflammatory indicator calprotectin in patients’ stool [7, 8]. Another study reported accumulation of cells expressing the lipopolysaccharide (LPS) receptor Toll-like Receptor 4 (TLR4) and cells immunoreactive for the T-cell marker cluster of differentiation (CD) 3 in colon tissue from PD patients [9]. Higher expression of genes encoding proinflammatory cytokines such as tumor necrosis factor (TNF), interferon gamma (IFNγ), interleukin (IL)-6, IL-1β, C-X-C motif ligand (CXCL) 8, and others in colonic biopsies [9, 10], and higher levels of IL-1α, IL-1β, CXCL8, and C-reactive protein (CRP) in stool have been found in PD patients compared to controls [11]. These immune mediators are associated with the canonical Nuclear Factor Kappa B (NFκB) signaling pathway, which is persistently activated in chronic inflammatory disorders [12] and appears to be similarly dysregulated in PD [13, 14].

Further evidence of the potential for excessive NFκB activity to contribute to parkinsonian neurodegeneration comes from rodent and cell culture models deficient in the Regulator of G-Protein Signaling 10 (RGS10) [15–17]. RGS10 is highly expressed in central and peripheral myeloid cells where it acts as a negative regulator of inflammation through inhibition of NFκB activity [15–18]. These studies have shown that RGS10 loss globally, in microglia, in macrophages, and in neurons increases susceptibility to immune- and oxidative stress-mediated dopaminergic neurodegeneration [15–17, 19, 20]. It is not known whether genetic RGS10 deficiency occurs in human populations, but it has been shown that RGS10 expression is suppressed in murine myeloid cells upon activation of TLR4 [15, 21]. It can be hypothesized, then, that individuals with chronic inflammatory conditions such as PD might have reduced RGS10 levels in activated immune cells, a condition which could contribute to the potentiation and persistence of proinflammatory responses and increase the likelihood of immune-mediated neurotoxicity. How RGS10 deficiency and the associated enhancement of NFκB signaling might affect PD-relevant gut-brain interactions has not previously been investigated.

Epidemiological studies have reported that individuals with inflammatory bowel disease (IBD), a condition characterized predominantly by inflammation of the distal small intestine and/or colon, are at increased risk for development of PD [22–25]. Research has also found that immunosuppressive treatment [26], and specifically anti-TNF therapy [23], significantly reduces IBD-associated PD risk. These findings support the theory that PD pathology could originate in the gut and strongly suggest that gut inflammation is a critical mediator of the association between IBD and PD. Meta-analysis indicates, however, that the increased risk of PD conferred by IBD is 28–30% [27], and some studies did not identify any increased risk [26] or suggested that the association could be accounted for by other factors such as surveillance bias [24]. Two studies observed an IBD-PD association primarily in men [22, 23], which could be driven by greater prevalence of PD in men [1], while other studies reported the opposite [24] or found no sex differences [25]. There is also overlap in genetic variants associated with PD and IBD [28, 29], and the disease risk conferred by PD-linked genetic polymorphisms can be impacted synergistically by environmental exposures [30]. Certainly, PD is a complex and heterogenous disorder which typically necessitates the convergence of multiple risk factors including age, sex, genetic predisposition, and environmental exposures. Investigating how these factors interact with GI pathology to impact neuron function is critical to understanding and managing any neurological risk posed by intestinal disease.

Research into the theory that gut inflammation contributes to the development of parkinsonian neurodegeneration is in its infancy. Several studies have reported that oral administration of the pesticide rotenone induces inflammation and microbiome alterations in the gut in addition to central neurodegeneration and motor deficits [31–35], and these neurological effects are mitigated in TLR4-deficient mice [9], implicating inflammatory responses, and this pathway in particular, as mediators of pathology. It has also been reported that male rats with dextran sodium sulfate (DSS)-induced colitis exhibited increased microglial activation, neuroinflammation, and neuron loss in the SNpc and that these effects diminished with depletion of peripheral macrophages [36]. Male mice given 2.5% DSS for seven days exhibited increased proinflammatory IL-1β in the SNpc but no neurodegeneration; a sub-chronic regimen of 3 weeks of 0.5% DSS was required to elicit dopaminergic neuron loss [37]. Another study detected no neuropathology in wild-type (WT) female mice after 12 weeks of 0.5% DSS, but in their transgenic female littermates overexpressing human mutant (A53T) α-synuclein, this DSS regimen was associated with neuroinflammation, greater accumulation of pathologic α-synuclein, neurodegeneration, and earlier age of onset for motor impairment compared to transgenic mice without colitis [38]. These studies suggest that active gut inflammation can influence the brain and even elicit neurodegeneration if sustained for a sufficient length of time, particularly in animals genetically predisposed to neurodegenerative disease. Significant questions remain, however, regarding the protracted effects of acute GI inflammation on the brain, the mechanisms that mediate them, and how they are influenced by interactions with sex and environmental risk factors.

To address these questions, we sought to identify inflammatory mediators active in colon biopsies from PD patients, and we investigated RGS10 levels in patients’ circulating immune cells. Based on our findings in humans, we utilized a mouse model of DSS-induced colitis, including male and female, WT and RGS10^−/−^ mice, as well as mice depleted of CD8+ T-cells, to examine the effects of gut inflammation and particular inflammatory mediators on the nigrostriatal system. We also assessed the extent to which colitis could exacerbate the effects of subsequent exposure to a known dopaminergic neurotoxicant [39] administered at subthreshold doses.

## Methods

### Human procedures

Human colon biopsies from the cohort described by Shannon et al. [40] were utilized in this study. PD patients (diagnosed according to United Kingdom Parkinson Disease Research Society Brain Bank criteria and without atypical or secondary parkinsonism, n = 6) were recruited from the Rush University Medical Center (RUMC) movement disorders clinic. Healthy controls (HCs, n = 6) matched to each PD subject were selected from the RUMC Department of Gastroenterology and Nutrition Tissue Repository based on age, sex, and body mass index. HCs had no history of gastrointestinal or neurological disease. Exclusion criteria for either group were diabetes, alcohol abuse, use of antiplatelet or anticoagulant drugs, primary gastrointestinal pathology, and any medical, neurological, or psychiatric condition not well-controlled. Subjects underwent unsedated limited unprepped flexible sigmoidoscopy to the distal sigmoid at approximately 20 cm from the anal verge. Cold biopsies were obtained using biopsy forceps from visually normal sigmoid colon tissue and were flash-frozen in liquid nitrogen and stored at −80 °C for 2 years until processing. Subject characteristics are provided in Additional file 1.

RGS10 levels were assessed in peripheral blood mononuclear cells (PBMCs) from the cohort described by Cook et al. [41]. PD patients (n = 33) and HCs (n = 13) were recruited through the Immune System and Neurological Disease research protocol at the Emory Movement Disorders Clinic. Participants completed a questionnaire and were excluded if they were younger than 40 years old or had neurologic, chronic infectious, or autoimmune comorbidities, and/or known familial PD mutations. All subjects in the final cohort were Caucasian with self-reported European ancestry. Blood was drawn by venipuncture, and PBMCs were isolated by density centrifugation over Ficoll-Paque (GE Healthcare) for flow cytometry.

### Mouse procedures

Mice were maintained on a 12 h:12 h light/dark cycle and provided standard chow ad libitum. RGS10^−/−^ mice generated as described by Lee et al*.* [16] were rederived at Emory University in a new Association for Assessment and Accreditation of Laboratory Animal Care (AAALAC)-certified specific pathogen-free facility on a C57BL/6 background.

Baseline genotype comparisons were made using 2-month-old male and female RGS10^+/+^ and RGS10^−/−^ littermates. For gut evaluations, mice were weighed, anesthetized by exposure to isofluorane (Piramal), and cervically dislocated. The colon was dissected, gently pressed and rinsed in PBS to remove feces, and its length measured. Distal colon tissue was frozen in optimal cutting temperature (OCT) compound for histology of frozen sections, fixed in 4% paraformaldehyde (PFA) overnight for whole mount histological preparations, or flash-frozen in liquid nitrogen for molecular assays and stored at −80 °C until processing. For colitis experiments, mice drank autoclaved tap water containing 2% DSS (Affymetrix) ad libitum for 5 days after which they were returned to autoclaved tap water. A subset of mice were cervically dislocated under isofluorane after 5 days on water, and distal colon tissue was flash-frozen for qPCR. In another set of mice, measurements of weight loss, feces consistency, and fecal blood were taken daily during DSS exposure and for 7 days after its removal and scored according to the criteria described in Additional file 2 to yield a daily disease activity index (DAI) for each mouse equal to the sum of the three scores. Occult blood was detected using Hemoccult II SENSA kits (Beckman Coulter) according to manufacturer’s protocol.

To evaluate the impact of colitis on central neuropathology, male and female 2–3-month-old RGS10^+/+^ and RGS10^−/−^ mice were assigned to one experimental group: H2O-Saline, H2O-MPTP, DSS-Saline, or DSS-MPTP (Additional file 3), with littermates divided across groups. Mice in H2O groups drank autoclaved tap water ad libitum. Mice in DSS groups underwent the regimen described above. Fifteen days after experiment initiation, mice in MPTP groups received a subacute regimen [42] of 18 mg/kg 1-methyl-4-phenyl-1,2,3,6-tetrahydropyridine (MPTP; Sigma-Aldrich) in saline subcutaneously (s.c.) (~ 100 µL) each day for 5 days. Mice in Saline groups received 100 μL s.c. sterile saline daily. Mice were coded by a researcher unaffiliated with the study so that the researchers conducting the experiments were blinded to genotype and treatment group until data analyses.

One day before experiment termination, ~ 200 μL blood from lancet puncture of the facial vein were collected into ethylenediaminetetraacetic acid (EDTA)-containing microfuge tubes. 100 μL were treated with 2 mL of 1X red blood cell lysis buffer (BioLegend) for 10 min at room temperature (RT) in the dark and then pelleted for flow cytometry, and the remainder was centrifuged to separate cells from plasma. Plasma was flash-frozen in liquid nitrogen and stored at −80 °C until processing for multiplexed immunoassay. The following day, mice intended for molecular tissue analysis were decapitated, and the SNpc and bilateral striatum brain regions were rapidly dissected on ice, flash-frozen in liquid nitrogen, and stored at −80 °C until processing. Mice intended for brain histology were given an i.p. injection of 40 μL Euthasol (Virbac), and, when unresponsive, were perfused with saline until no visible blood remained then with ice-cold 4% PFA in PBS for 5 min. The whole brain was then post-fixed in 4% PFA overnight followed by storage in 30% sucrose in PBS solution at least 24 h before cryosectioning for immunostaining.

CD8^+^ T-cell depletion was achieved in a separate cohort of mice by delivering 100 μg (2.5 μg/mL) anti-CD8b (BioXCell BE0223 clone 53–5.8) i.p. 3 days and 1 day prior to the initiation of the DSS-Saline or H2O-Saline regimen and every 7 days thereafter until the experiment endpoint (Additional file 4).

### RNA and protein isolation

The left hemisphere striata were manually homogenized in radioimmunoprecipitation assay (RIPA) buffer (150 mM NaCl, 1% Triton X-100, 0.1% sodium dodecylsulfate [SDS], 50 mM Tris HCl) with cOmplete Protease Inhibitor cocktail (Roche, 1 tablet/40 mL buffer) using an electric pestle. SNpc and colon tissue were homogenized in cold TRIzol reagent (Life Technologies) using a TissueLyser II (Qiagen; 2 cycles, 2 min each, 20 Hz) and a 5 mm stainless steel bead (Qiagen) to isolate RNA and protein. RNA was isolated using QIAshredder and RNeasy mini kits (Qiagen) according to manufacturer’s protocol, and concentrations were measured using a NanoDrop 2000 spectrophotometer (Thermo Fisher). Protein was isolated from the organic phase of the Trizol preparation by precipitation in methanol and centrifugation followed by resuspension in RIPA buffer with cOmplete Protease Inhibitor cocktail. Protein concentrations were measured using the BCA Protein Assay Kit (Pierce) according to manufacturer’s protocol. RNA was purified with DNAse I (Life Technologies; 0.64μL per 4 μg) for 30 min at 37 °C, then 10 min at 75 °C, and converted to cDNA using SuperScript II Reverse Transcriptase (Life Technologies), deoxyribonucleotide triphosphates (dNTPs) (Life Technologies), and random hexamer primers (Integrated DNA Technologies) according to manufacturer’s protocol for the enzyme and as published previously [43].

### Western blot

Protein homogenates (10 μg, 20 μg for RGS10 measurements) in Laemmli buffer (Bio-Rad) were resolved on 4–20% Mini-PROTEAN TGX Precast gels (Bio-Rad) and transferred to membranes using the Trans-Blot Turbo System (Bio-Rad) according to manufacturer’s protocol. Total protein was visualized using the REVERT Total Protein Stain (LI-COR) according to manufacturer’s protocol and imaged on an Odyssey Fc 2800 (LI-COR). Blots were then cut to facilitate single exposure to multiple antibodies, washed, blocked for ~ 1 h, and incubated overnight with primary antibodies in 5% powdered milk in Tris-buffered saline with 0.1% Tween-20 (TBST). Blots were washed and incubated one hour with appropriate horseradish peroxidase (HRP)-conjugated secondary antibodies (Additional file 5). Chemiluminescent signal was imaged on Azure Biosystems’ C400 system. Bands were quantified using Image Studio Lite v5.2. Gut protein levels were normalized to levels of β-actin in each sample from the same blot. β-actin was deemed an inappropriate loading control for striatum samples because its levels were not consistent across experimental groups, so levels of targets measured in striata were normalized to total protein. Full, unedited images of all blots are provided in Additional file 6.

### Quantitative polymerase chain reaction (qPCR)

qPCR was performed as described previously [44] with minor modifications. For each qPCR reaction, 25 ng cDNA was run in triplicate with SYBR Green PCR Master Mix (Life Technologies) and 150 nM validated forward and reverse oligonucleotide primers (Integrated DNA Technologies) (Additional file 7) on an ABI Prism 7900 HT Fast Real-time PCR System (Applied Biosystems). Averaged triplicate target gene cycle threshold (Ct) values were normalized to the averaged values of two housekeeping genes, *RNA18SN5* and cyclophilin (*PPIA*) for human samples and *Gapdh* and *Ppia* for mouse samples. Because Ct values are inversely associated with gene expression, for intuitive interpretation, relative mRNA expression was calculated by subtracting normalized Ct values from a standard number (19).

### High performance liquid chromatography (HPLC)

Levels of dopamine and related analytes in the right hemisphere striatum were evaluated by HPLC as described previously [45] with the following specifications: Tissue was sonicated (Tissue-Tek, output 3, duty cycle 30%) in 20 × volume of 100 mM perchloric acid and transferred to 0.45 μm polyvinylidene difluoride (PVDF) spin-filter (Grace Davison Discovery Science). Levels of dopamine (DA), 3,4-dihydroxyphenylacetic acid (DOPAC), homovanillic acid (HVA), and L-3,4-dihydroxyphenylalanine (L-DOPA) were quantified by comparing peak areas to standards. As L-DOPA was undetectable in the majority of samples, it was not analyzed further.

### Multiplexed immunoassays

Distal colon tissue was homogenized in buffer (125 mM Tris, 15 mM MgCl2, 2.5 mM EDTA pH 7.2, 1% Triton X-100, 1 tablet cOmplete protease inhibitors [Roche] per 10 mL buffer) using a TissueLyser II (Qiagen; 2 cycles, 2 min each, 20 Hz) and a 5 mm stainless steel bead (Qiagen). Remaining solids were pelleted and supernatants collected. Protein concentrations in supernatants were determined using the BCA Protein Assay Kit (Pierce) according to manufacturer’s protocol and adjusted to 7 μg/μL with homogenization buffer. Colon supernatants and plasma were diluted 1:1 with homogenization buffer immediately before measuring cytokines with the V-PLEX Proinflammatory Panel 1 Mouse Kit (Meso Scale Discovery; MSD) on the MSD QuickPlex instrument.

### Flow cytometry

PBMCs were washed with PBS, resuspended in PBS, and transferred to a V-bottom plate (Corning). They were incubated 30 min at RT in the dark in 50 μL LIVE/DEAD Fixable Aqua (mouse PBMCs) or Red (human PBMCs) Dead Cell Stain (Life Technologies) prepared according to manufacturer’s protocol and diluted 1:2000 in PBS.

Mouse PBMCs were washed in PBS, then incubated 20 min on ice in 50 μL solution containing Fc-blocking anti-CD16/CD32 and 9 anti-mouse fluorophore-conjugated antibodies (Additional file 5) in fluorescence-activated cell sorting (FACS) buffer (1 mM EDTA and 0.1% sodium azide in PBS). Stained PBMCs were washed in FACS buffer, then incubated 30 min on ice with 1% PFA.

Human PBMCs were washed in PBS, treated with Human TruStain FcX Receptor Blocker (Biolegend) according to manufacturer’s protocol, then incubated 20 min on ice in 50 μL of FACS buffer containing 8 anti-human fluorophore-conjugated antibodies (Additional file 5). Cells were washed with FACS buffer, and then intracellular staining was performed using the Fixation and Permeabilization Kit (Invitrogen). PBMCs were incubated with 50μL Reagent A (Fixation Medium) for 15 min at RT, washed with FACS buffer, then incubated with 50 μL Reagent B (Permeabilization Medium) and anti-RGS10 antibody (Santa Cruz Biotechnology) for 20 min at RT. Cells were washed with FACS buffer, and the last step was repeated with anti-goat IgG PerCP-Cy5.5-conjugated secondary antibody (Santa Cruz Biotechnology) (Additional file 5).

After staining, mouse and human PBMCs were washed and resuspended in FACS buffer, and 10μL AccuCheck Counting Beads (Thermo Fisher) were added to each sample before evaluation by LSR II flow cytometer and FACSDiva software (BD Biosciences). Results were analyzed using FlowJo 10.4.2 according to the gating strategies in Additional files 8 and 9. Frequencies and counts of each cell population and geometric mean fluorescence intensities (GMFIs) of markers were assessed. Cell counts for mice were calculated according to the manufacturer’s protocol for 100 μL blood.

### Immunostaining

For staining frozen gut sections, 7 μm sections of rolled distal colon tissue from RGS10^+/+^ and RGS10^−/−^ mice were cut from frozen OCT blocks and mounted on slides, then fixed in 4% PFA for 10 min prior to staining. Tissue for gut whole mounts was prepared by dissecting the muscle and myenteric plexus layer of fixed distal colon tissue from the rest of the tissue containing the lamina propria under a dissecting microscope. 40 μm sections of SNpc were cut on a freezing microtome from fixed brains and rinsed in PBS.

Brain tissue intended for brightfield microscopy was incubated in 3% hydrogen peroxide for 15 min at 37 °C to block endogenous peroxidase activity. All tissue was blocked 1 h at RT in PBS with 0.3% Triton X-100 (EK Industries) and 5% Normal Serum (NS; Jackson ImmunoResearch) from the host species of the secondary antibody, then incubated in primary antibodies for 1 h at RT for frozen sections and overnight at 4 °C for whole mounts and brain sections. Tissue was rinsed thoroughly and then incubated for 1 h at RT with biotinylated or fluorophore-conjugated secondary antibodies. Antibodies were diluted in PBS with 0.3% Triton X-100 and 1% NS sections and are detailed in Additional file 5. Brain sections were washed in PBS and transferred to avidin–biotin-peroxidase complex (ABC) (Vector Laboratories) solution for 1 h at 4 °C, then treated using 3,3'-diaminobenzidine (DAB) Tablets (Sigma-Aldrich) according to manufacturers’ protocols. After washing with PBS, gut tissue was transferred to microscopy slides and treated with VECTASHIELD mounting medium containing DAPI (Vector Laboratories). Images were obtained on a Nikon Eclipse 90i microscope with a Nikon DS-Qi1MC camera and Nikon NIS Elements software.

### Statistics

Two-tailed t-test was used to compare HC and PD colonic biopsy data, HC and PD PBMC data, age, and intake of caffeine, tobacco, and non-steroidal anti-inflammatory drugs (NSAIDs). Ordinal regression was used to compare gender distribution in HC and PD groups and was performed in R [46] using RStudio [47] and packages “ordinal” [48] and “car” [49]. Two-way Analysis of Variance (ANOVA) with Sidak’s post hoc was used to compare data by genotype collected from male and female mouse colon samples. Two-way repeated measures ANOVA with Sidak’s post hoc was used to compare DAI of RGS10^+/+^ and RGS10^−/−^ mice over the course of DSS colitis. Two-way ANOVA with Tukey’s post hoc was used for all other genotype and treatment comparisons, with male and female mice evaluated separately. Pearson correlations were used to assess associations between variables. Evaluation of interactions between CD8^+^ T-cell depletion status and DSS exposure was conducted using generalized linear models and performed in performed in R [46] and RStudio [47] using the “stats” package [46]. Unless otherwise specified, statistical analyses were performed using GraphPad Prism 6, *p* < 0.05 was considered significant, and data are represented as mean ± SEM.

Supervised machine learning using a random forest ensemble was employed to rank the measures in this study according to the value of their contribution to a regression model of striatal tyrosine hydroxylase (TH) levels. 34 variables were included in the model. Data on levels of phosphorylated TH, *Th* mRNA, dopamine transporter (DAT), vesicular monoamine transporter 2 (VMAT2), and DA and its metabolites were excluded as they were known to be directly related to striatal TH protein levels. Separate models were built for male and female mice with 55 individuals included for each. Due to this relatively small sample size, the data were not partitioned, and models were trained on the entire dataset to generate descriptive information. Three hundred decision trees were built, and nine variables were considered for partitioning in each tree. These models were developed in R [46] using RStudio [47] and the graphic user interface rattle [50] with packages “rattle” [50] and “magrittr” [51].

## Results

### Indicators of inflammation and immune cells are increased in colon tissue from PD patients

**Fig. 1:**
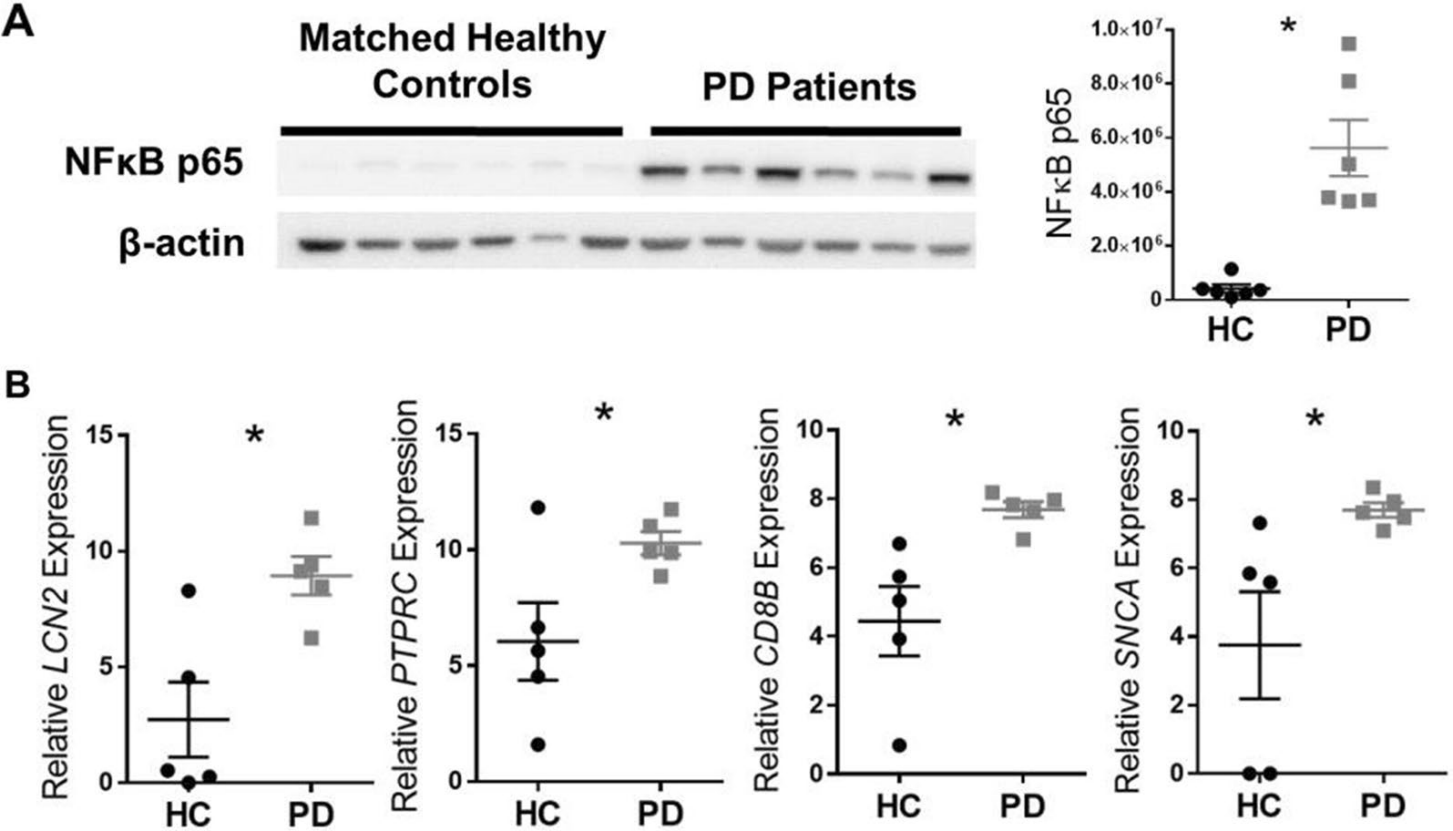
Colonic inflammation is evident in PD patients. In sigmoid colon biopsies from PD patients and individually matched healthy controls (HC), **A** NFκB p65 protein (n = 6, paired t-test, two-tailed, *p* = 0.0052) and **B** relative mRNA levels (n = 5, paired t-test, two-tailed, *p* < 0.05 for all except *SNCA* for which *p* = 0.07). * indicates significant differences between groups

To expand upon reports of gastrointestinal inflammation in PD, we assessed mediators of inflammation in sigmoid colon biopsies from PD patients and matched HCs. We observed a marked increase in NFκB p65 protein levels in the colon of PD patients compared to HCs (*p* = 0.0006) (Fig. 1A). Expression of *LCN2*—a common indicator of gut inflammation, *PTPRC* which encodes CD45—an immune cell marker,*CD8B*—a cytotoxic T-cell marker, and *SNCA* which encodes α-synuclein—a protein involved in PD pathology—was higher than in HCs (*p* = 0.0093, *p* = 0.0413, *p* = 0.0140, *p* = 0.0366, respectively) (Fig. 1B).

### RGS10 levels are reduced in peripheral blood immune cells of PD patients

**Fig. 2:**
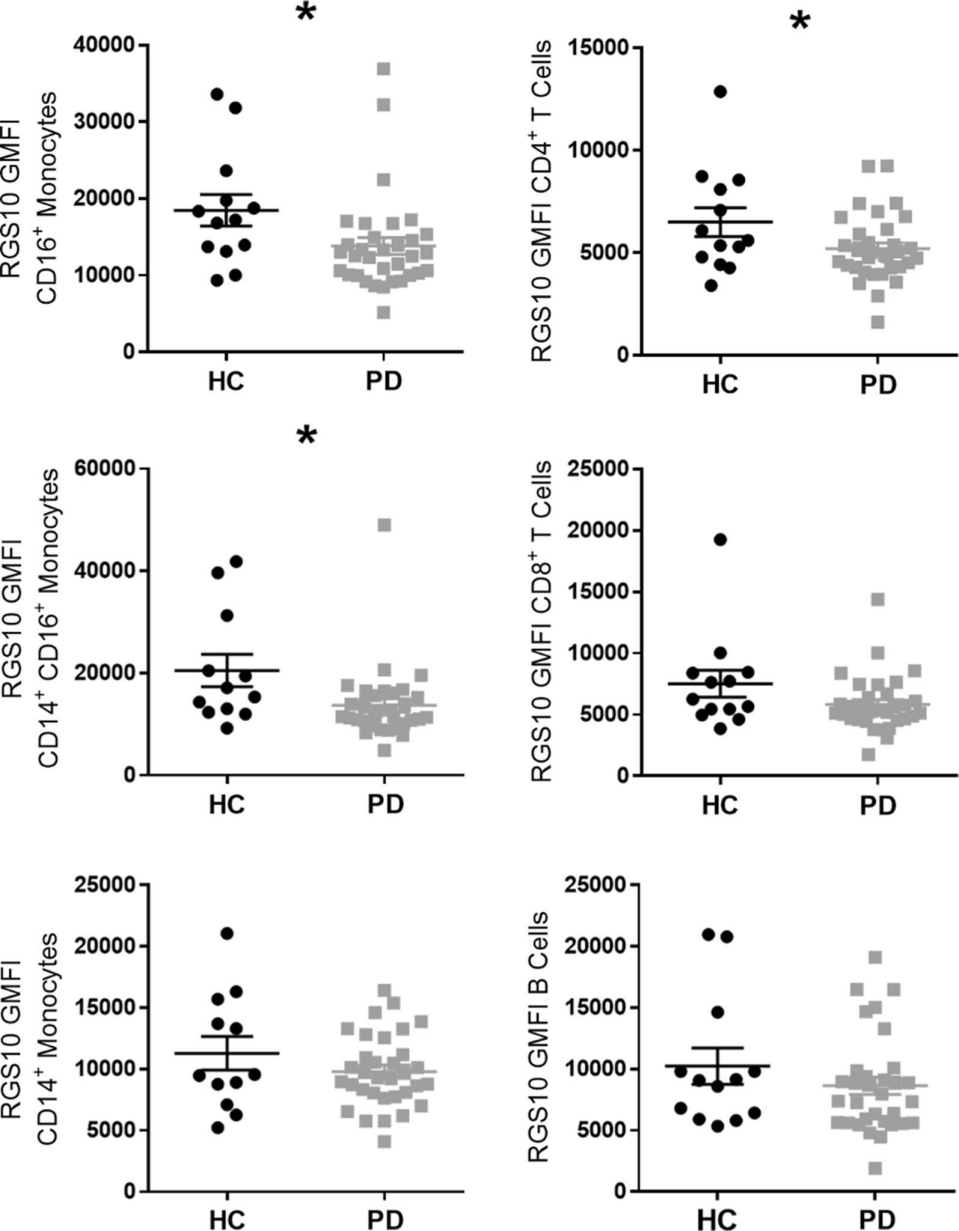
RGS10 levels reduced in peripheral blood CD16+ and CD14+ CD16+ monocytes and CD4+ T-cells in PD. RGS10 levels (geometric mean fluorescence intensity; GMFI) measured by flow cytometry in peripheral blood mononuclear cells (PBMCs) from PD patients and HC (n = 12–33, t-test, two-tailed). * indicates significant (*p* < 0.05) differences between groups

To evaluate the potential for heightened NFκB activity systemically in PD, we measured RGS10 levels (GMFIs) in PBMCs from a larger cohort of PD patients and HCs. RGS10 levels were highest in monocytes expressing CD16 and lowest in T-cells (Fig. 2). In CD16^+^ monocytes, CD14^+^ CD16^+^ monocytes, and CD4^+^ T-cells, RGS10 levels were significantly lower in PD patients compared to HCs (*p* = 0.043, *p* = 0.0223, *p* = 0.0409, respectively) (Fig. 2). There were no significant differences between the PD and HC groups in age, gender, or intake of caffeine, tobacco, or NSAIDs (Additional file 10).

### RGS10 deficiency induces intestinal inflammation and dysfunction in mice

**Fig. 3:**
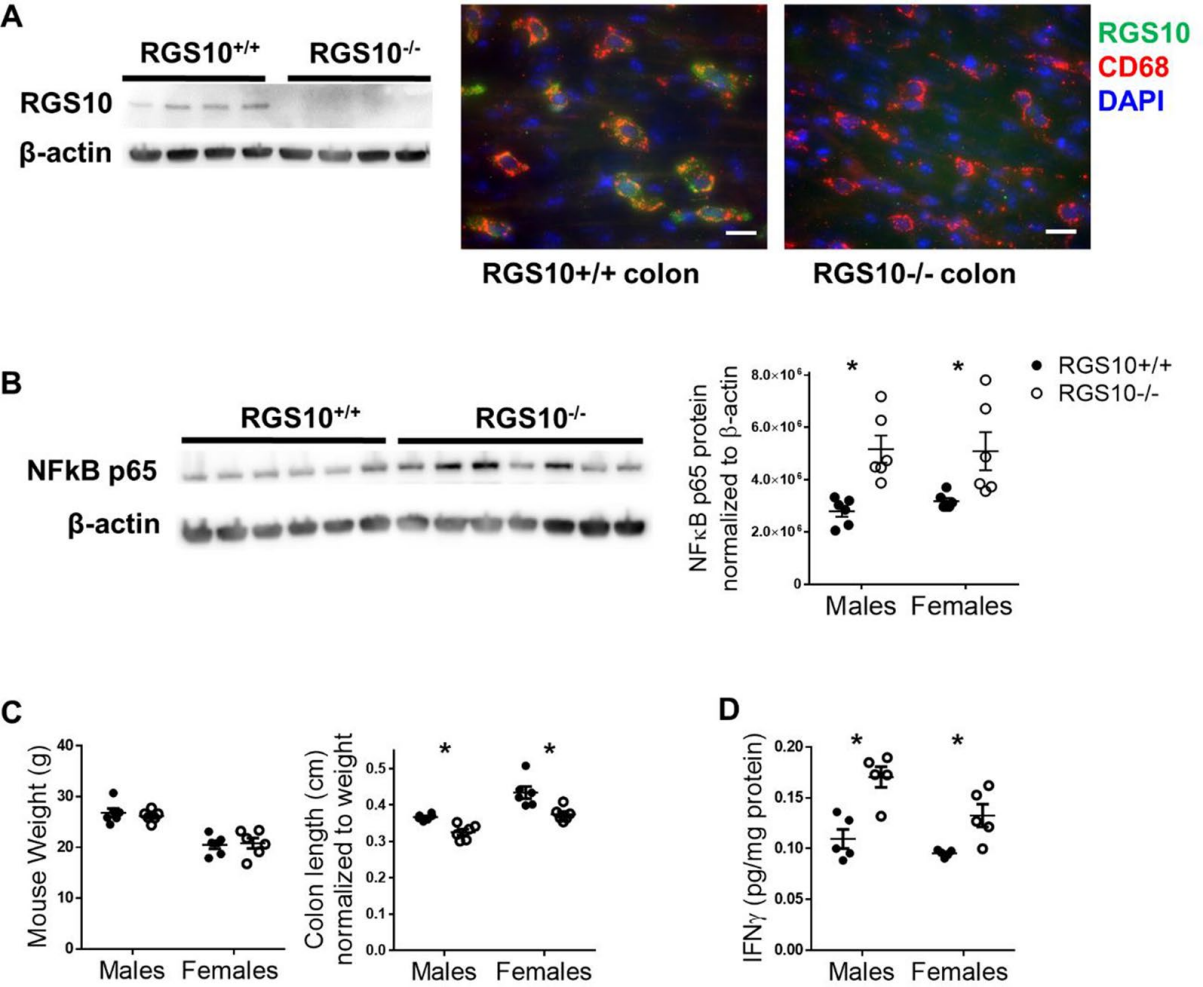
RGS10 deficiency induces intestinal inflammation and dysfunction in mice. In 2-month-old male and female RGS10^+/+^ and RGS10^−/−^ littermates: **A** RGS10 protein and RGS10^+^ cells (green) which are also CD68^+^ (red) in colon tissue counterstained with DAPI (40x, scale bar = 20 μm). **B** NFκB p65 protein in distal colon tissue (two-way ANOVA, Sidak’s post hoc*,* n = 6 per sex, genotype effect *p* = 0.0002). **C** Mass and colon length normalized to mass (n = 6 per sex, for colon length: genotype effect *p* < 0.0001, sex effect *p* < 0.0001), **D** IFNγ concentrations and (n = 5 per sex, genotype effect *p* < 0.0001, sex effect *p* = 0.0104). Blots are representative images containing samples from both male and female mice. * indicates significant differences between genotypes

RGS10^+/+^ and RGS10^−/−^ mice were used to explore the role of RGS10 in the intestinal environment. RGS10-expressing cells were present in the murine colon and were primarily of myeloid rather than lymphocyte or neuronal lineage (Fig. 3A, Additional file 11). Evidence of inflammation in the colon of RGS10^−/−^ mice was also observed. Consistent with previous findings regarding RGS10’s inhibition of NFκB [16, 17], NFκB p65 levels were significantly increased in the colon of mice lacking RGS10 (males *p* = 0.0033, females *p* = 0.0167) (Fig. 3B). While there was no significant difference in body weight between RGS10^+/+^ and RSG10^−/−^ mice, the colon lengths of RGS10^−/−^ mice normalized to body weight were shorter than their WT littermates (males *p* = 0.0179, females *p* = 0.0010) (Fig. 3C), and levels of the proinflammatory cytokine IFNγ were significantly increased in RGS10^−/−^ colon tissue (males *p* = 0.0004, females *p* = 0.0191) (Fig. 3D). No significant differences were found in levels of IL-10, IL-12 p70, IL-1β, IL-2, IL-4, IL-5, IL-6, CXCL1, or TNF (data not shown).

### Inflammatory features of DSS colitis overlap with those found in colon tissue from PD patients

**Fig. 4:**
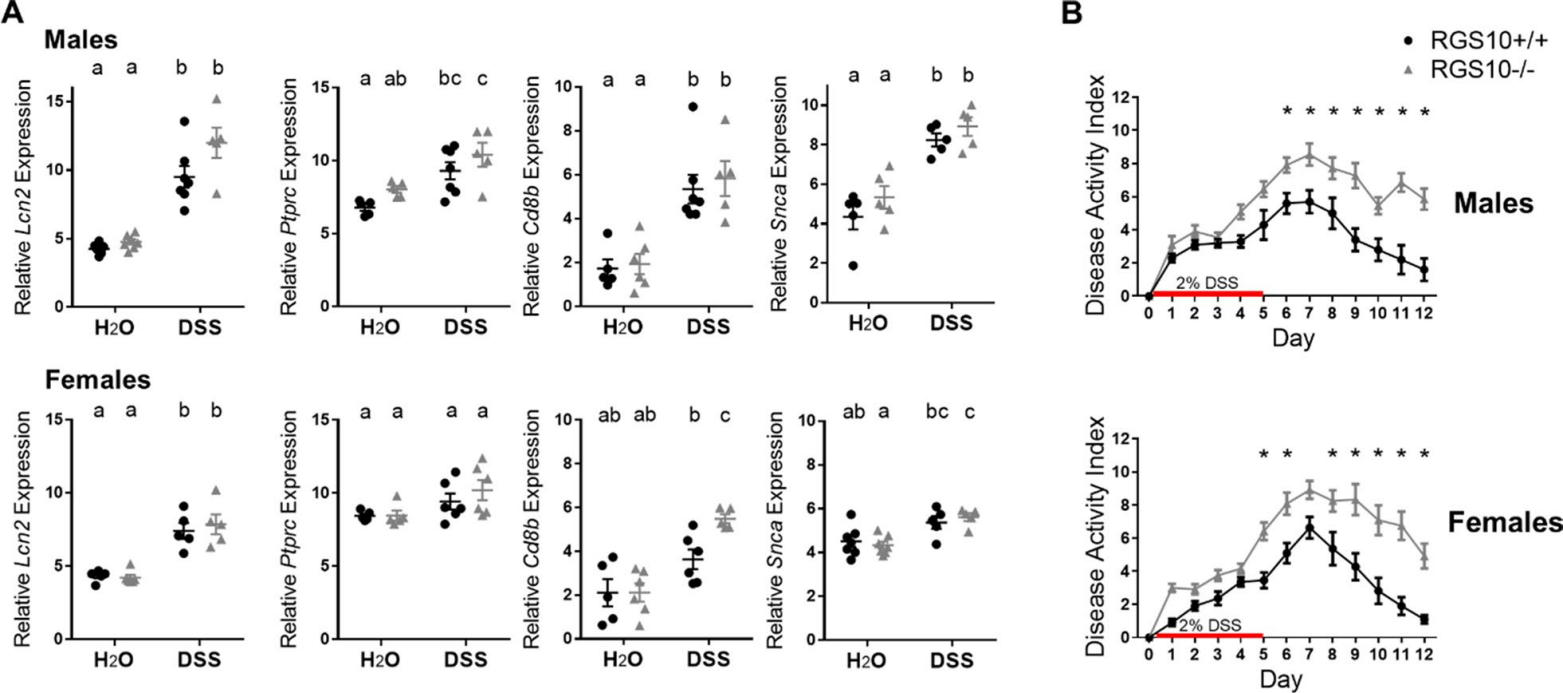
DSS colitis induces intestinal inflammation, and loss of RGS10 worsens colitis. **A** Relative mRNA levels in distal colon tissue from mice given H_2_O or 2% DSS (5d) followed by water (5d) (n = 5–7 per group (groups distinguished by sex, genotype, and treatment), two-way ANOVA, treatment effect *p* < 0.05 for all, genotype effect insignificant except for male *Lcn2* and *Ptprc* for which *p* < 0.05, Tukey’s post hoc). Letter(s) centered above groups reflect results of post hoc tests. Groups that do not share any letter are significantly different from one another. **B** Disease activity indices of mice with DSS colitis (n = 10–12 per group (groups distinguished by sex, genotype, and treatment), two-way repeated measures ANOVA, genotype effect *p* = 0.0002 for males and *p* < 0.0001 for females, Sidak’s post hoc). * indicates significant differences between genotypes each day by post hoc tests

DSS colitis is commonly used to model aspects of IBD [52]. To evaluate its impact on the inflammatory pathways that were upregulated in the PD colon, we measured the murine equivalents of those inflammatory indicators in distal colon tissue from RGS10^+/+^ and RGS10^−/−^ mice. Acute DSS colitis significantly increased expression of *Lcn2*, *Ptprc*, *Cd8b*, and *Snca* in the colon of male mice and of *Lcn2* in female mice. Expression of *Cd8b* and *Snca* increased with DSS only in RGS10^−/−^ and not RGS10^+/+^ females. A significant effect of genotype on *Lcn2* (*p* = 0.0300) and *Ptprc* (*p* = 0.0495) expression was also observed for male mice, with higher average levels present in RGS10^−/−^ tissue (Fig. 4A). A more pronounced genotype impact was seen in the DAI of DSS colitis; male (*p* = 0.0006) and female (*p* < 0.0001) RGS10^−/−^ mice developed more severe and more persistent colitis than WT mice (Fig. 4B).

### Colitis and RGS10 deficiency perturb nigrostriatal dopaminergic systems and augment effects of MPTP

To evaluate persistent effects of colitis on the brain and potential interactions with other factors such as sex, heightened NFκB-associated inflammatory responses, and exposure to neurotoxicants, male and female RGS10^+/+^ and RGS10^−/−^ mice underwent DSS colitis (Additional file 12) followed by subacute dosing with MPTP. Five weeks after the last exposure to DSS and three weeks after the last exposure to MPTP, SNpc and striatum of the mice in four experimental groups (H2O-Saline, H2O-MPTP, DSS-Saline, DSS-MPTP) were evaluated for indications of neuropathology (Additional file 3).

**Fig. 5:**
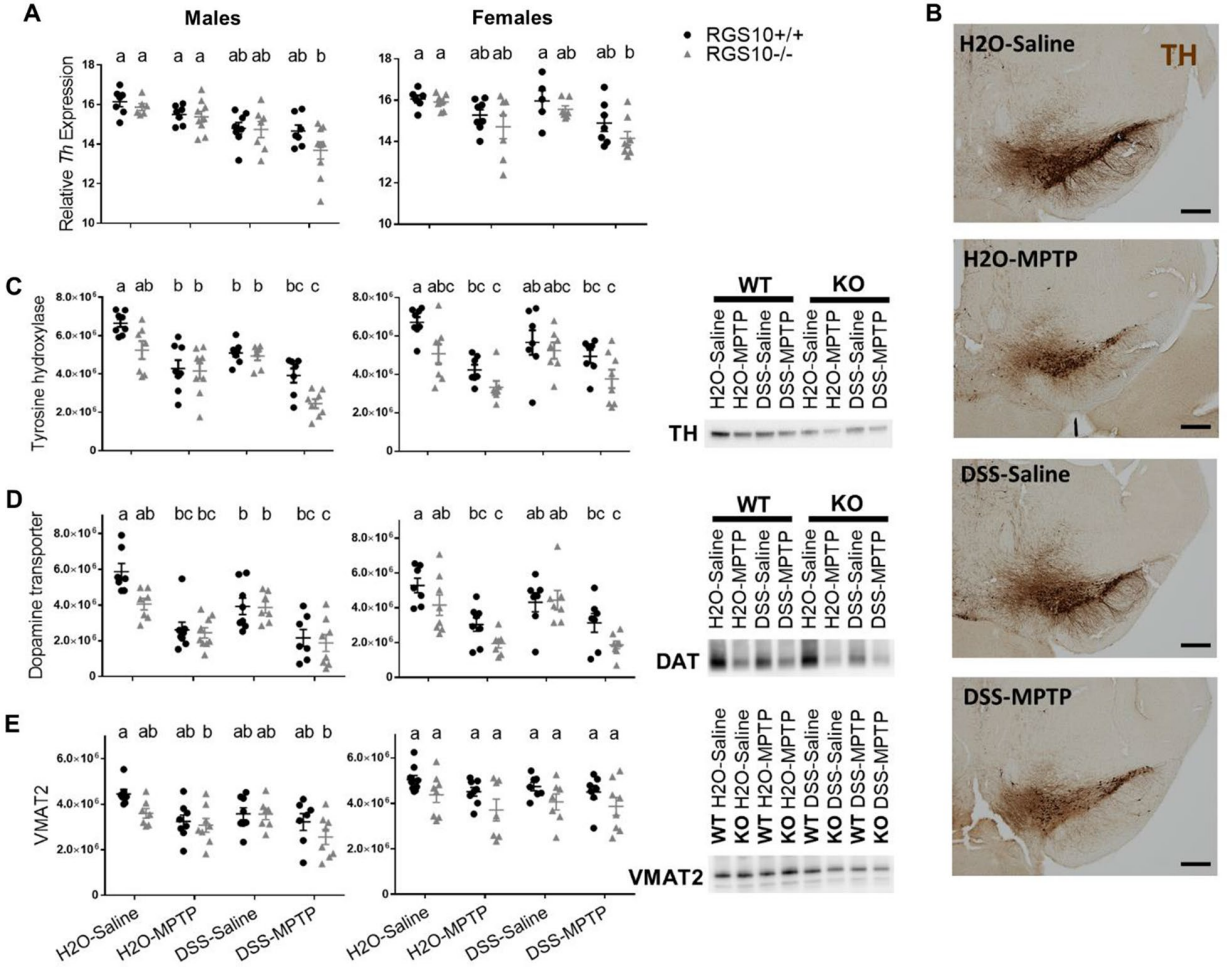
DSS colitis and RGS10 deficiency impact nigrostriatal dopamine pathways and increase susceptibility to MPTP. **A** Relative mRNA levels encoding tyrosine hydroxylase (TH) in SNpc (n = 5–8 per group (groups distinguished by sex, genotype, and treatment), two-way ANOVA, treatment effect *p* < 0.001, Tukey’s post hoc), **B** representative images of TH^+^ (brown) cells in the RGS10^+/+^ SNpc (4 × magnification, scale bar = 250 μm), and **C** TH, **D** dopamine transporter, and **E** VMAT2 protein in striatum measured by western blot and normalized to total protein (n = 7–9 per group (groups distinguished by sex, genotype, and treatment); two-way ANOVA, treatment effect *p* < 0.01 for all except VMAT2 for females, genotype effect *p* < 0.05 for all, Tukey’s post hoc) from mice given H_2_O or DSS followed by saline or MPTP. Letter(s) centered above groups reflect results of post hoc tests. Groups that do not share any letter are significantly different from one another

In the SNpc in both sexes, the combination of DSS colitis, neurotoxic insult from MPTP, and genetic susceptibility caused by the loss of RGS10 were necessary to produce significant reductions in levels of mRNA encoding TH—the enzyme which catalyzes the rate-limiting step in the synthesis of dopamine (Fig. 5A). While nigral *Th* expression measured by qPCR correlated significantly with striatal TH protein measured by western blot (males *p* = 0.0002, females *p *= 0.0004) (Additional file 13A), protein levels of TH were more sensitive to experimental manipulations. TH protein levels were significantly reduced by MPTP in males and females as well as by colitis alone in male mice compared to RGS10^+/+^ H2O-Saline levels (Fig. 5C). A similar pattern was observed in the abundance of TH protein in the SNpc by immunostaining (Fig. 5B). A significant effect of genotype (males *p* = 0.0015, females *p* = 0.0012) on striatal TH levels was also observed, with slightly lower levels in RGS10^−/−^ mice. The lowest levels of striatal TH were measured in RGS10^−/−^ DSS-MPTP males, and TH in this group was significantly reduced compared to males exposed to DSS or MPTP alone (Fig. 5C).

Levels of DAT and VMAT2, which regulate dopamine signaling, were found to correlate with each other (*p* = 0.0002) and with TH (*p* < 0.0001) (Additional file 13B). Patterns in DAT levels in the striatum across treatment groups closely resembled those of TH (Fig. 5D). Striatal VMAT2 levels typically do not diminish markedly unless substantial degeneration of nerve terminals has occurred [53], and we observed significant reductions in VMAT2 only in RGS10^−/−^ MPTP-treated male mice compared to RGS10^+/+^ H2O-Saline mice (H2O-MPTP *p* = 0.0231, DSS-MPTP *p* = 0.0004) (Fig. 5E). These data suggest that, while a single episode of colitis does impact the health of dopaminergic neurons in male mice, it is not sufficient to induce pronounced neurodegeneration of the nigrostriatal pathway.

### Increased activity of TH following colitis may prevent dopamine deficiency

**Fig. 6:**
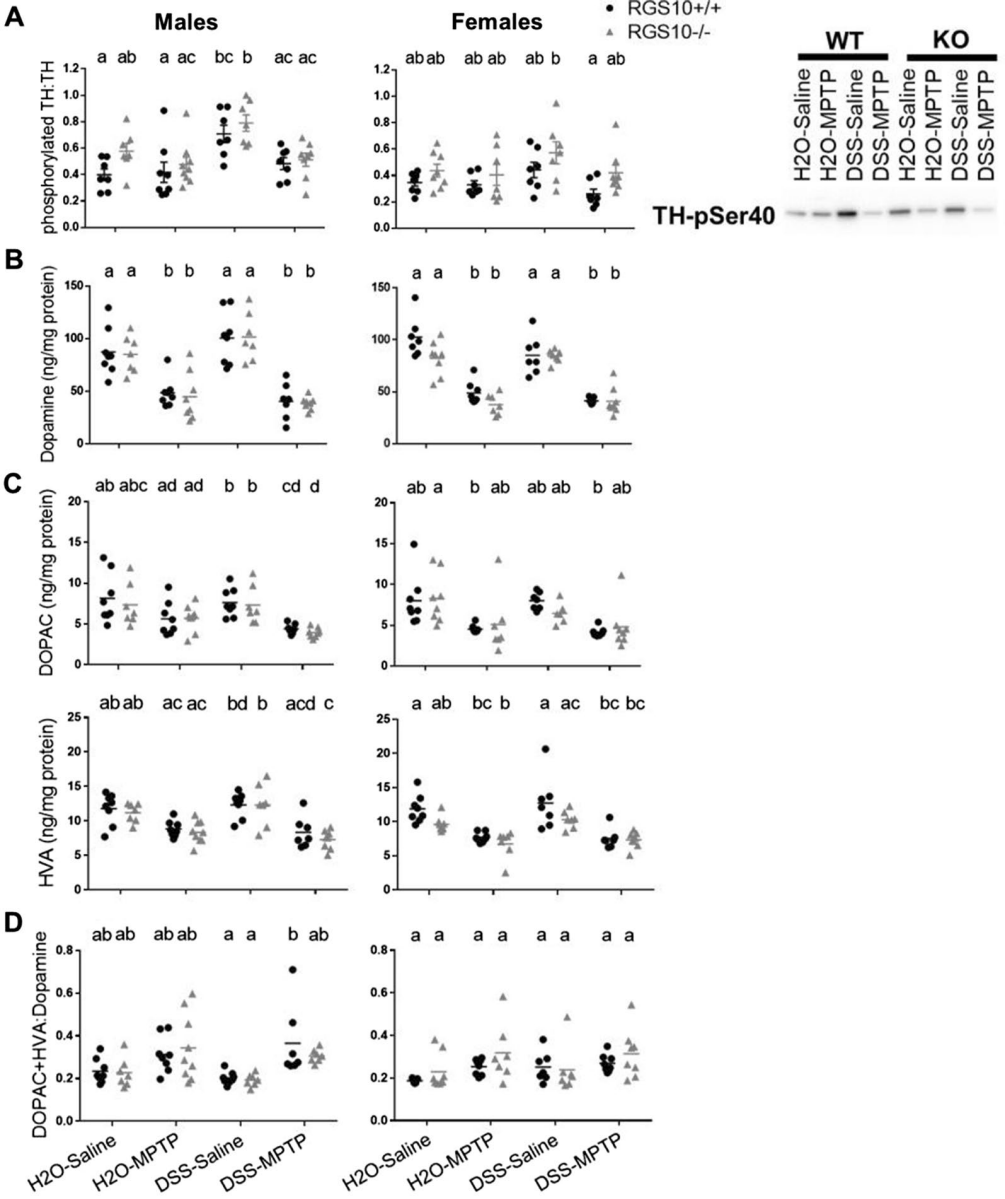
Increased tyrosine hydroxylase activity after colitis may prevent dopamine deficiency. **A** Ratio of TH protein phosphorylated at serine 40 to total TH measured by western blot (n = 7–9 per group (groups distinguished by sex, genotype, and treatment), two-way ANOVA, treatment effect and genotype effect *p* < 0.05, Tukey’s post hoc). **B** Levels of dopamine, **C** 3,4-dihydroxyphenylacetic acid (DOPAC), homovanillic acid (HVA), and **D** the ratio of the sum of DOPAC and HVA to dopamine measured by HPLC in striatum from mice given H_2_O or DSS followed by saline or MPTP (n = 6–9 per group (groups distinguished by sex, genotype, and treatment), two-way ANOVA, treatment effect *p* < 0.05 for all, genotype effect *p* < 0.05 for dopamine and HVA in females, Tukey’s post hoc). Letter(s) centered above groups reflect results of post hoc tests. Groups that do not share any letter are significantly different from one another

A lack of colitis-induced nigrostriatal neurodegeneration was supported by measurements of phosphorylated TH as well as DA and its metabolites DOPAC and HVA. Phosphorylation of TH at serine 40 (TH-pSer40) enhances the enzyme’s activity [54], and the ratio of TH-pSer40 to total TH indicates the proportion of the enzyme that is in this active state. Both genotype (males *p* = 0.0423, females *p* = 0.0054) and treatment (males *p* < 0.0001, females *p* = 0.0243) affected striatal TH-pSer40:TH ratios in this study. RGS10^−/−^ mice exhibited slightly higher TH-pSer40:TH ratios than RGS10^+/+^ mice, and the highest ratios were found in DSS-Saline mice. (Fig. 6A).

Accordingly, while MPTP-treated mice of both sexes and genotypes exhibited significant reductions in striatal DA compared to saline-dosed control mice (Fig. 6B), DSS treatment did not significantly impact levels of DA or its metabolites (Fig. 6B, C). MPTP only produced slight and inconsistent reductions in DOPAC and HVA compared to saline-treated groups (Fig. 6C). Under conditions in which the nigrostriatal pathway is degenerating, the ratio of levels of DOPAC and HVA to DA can increase as inadequate DA supplies are more rapidly metabolized. While we found a significant main effect of treatment (males *p* = 0.0002, females *p* = 0.0338) on the ratio of DA metabolites to DA, differences among groups by post hoc tests were minimal (Fig. 6D). There was a significant main effect of genotype on levels of DA (*p* = 0.0214) and HVA (*p* = 0.0048) in females with slightly lower levels in RGS10^−/−^ mice (Fig. 6B, C).

### RGS10 deficiency impacts peripheral blood immune cell populations

**Fig. 7:**
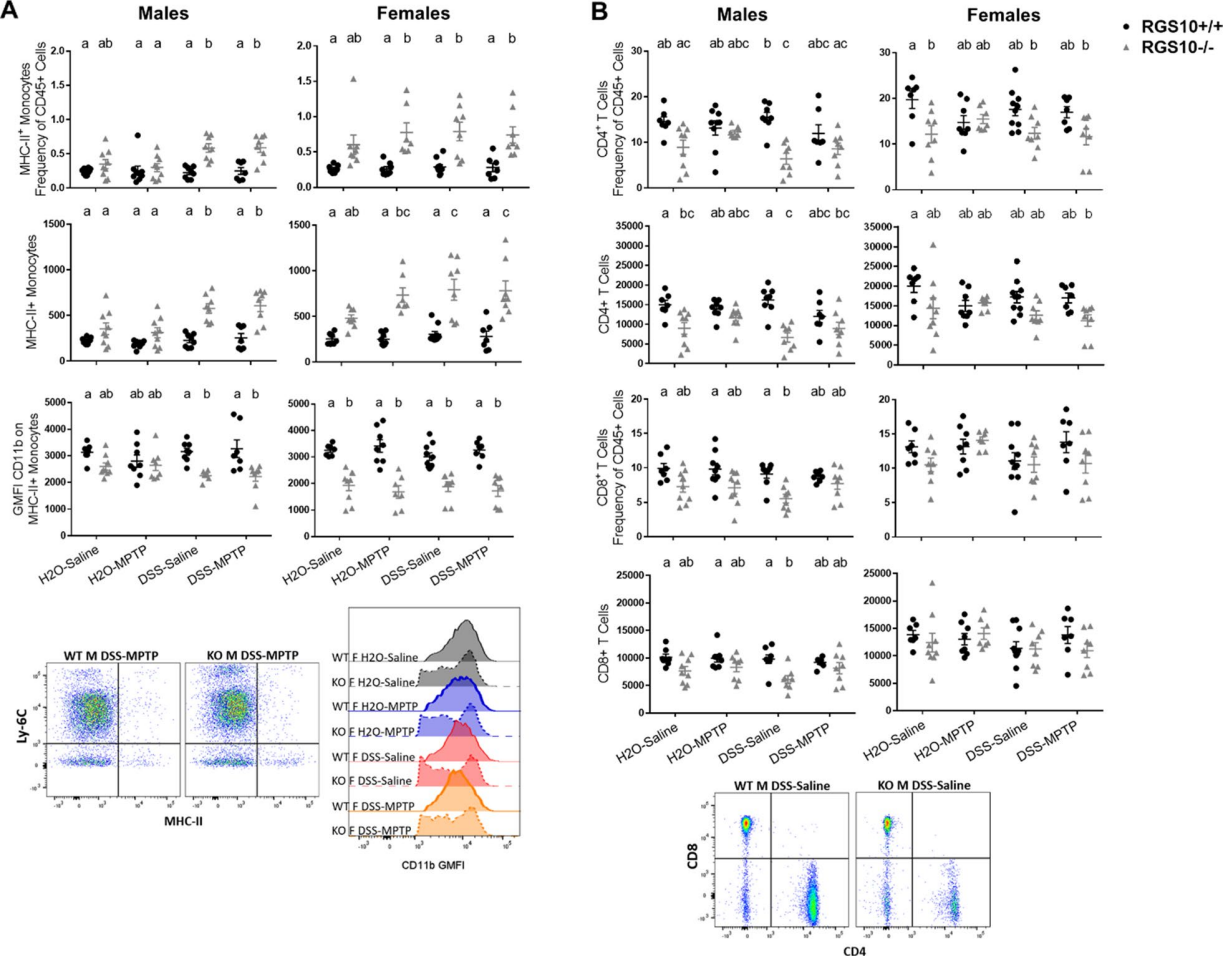
RGS10 deficiency impacts circulating non-classical monocytes and T-cells, and colitis augments these effects. **A** Frequency of CD45^+^ cells, counts, and GMFI of CD11b for Ly-6C^−^ MHC-II^+^ (non-classical) monocytes and **B** frequency of CD45^+^ cells and counts of CD4^+^ and CD8^+^ T-cells in PBMCs from mice given H_2_O or DSS followed by saline or MPTP determined at endpoint by flow cytometry (n = 7–10 per group (groups distinguished by sex, genotype, and treatment), two-way ANOVA, genotype effect *p* < 0.05 for all except female CD8^+^ T-cells, treatment effect *p* < 0.05 for male MHC-II^+^ monocyte frequency and male and female MHC-II^+^ monocyte counts, interaction *p* < 0.05 for male MHC-II + monocytes and male CD4 + T-cells frequency and counts, Tukey’s post hoc). Letter(s) centered above groups reflect results of post hoc tests. Groups that do not share any letter are significantly different from one another

To assess whether modulation of dopaminergic activity in the brain weeks after colitis might be the result of persistent systemic immune activation, PBMCs were analyzed by flow cytometry at the experiment endpoint. The effects observed were predominantly driven by genotype rather than treatment. Among DSS-treated males and DSS- and/or MPTP-treated females, RGS10^−/−^ mice had more Ly-6C^−^ MHC-II^+^ non-classical monocytes in circulation compared to RGS10^+/+^ both as a frequency of total immune cells and by absolute counts (Fig. 7A). In RGS10^−/−^ DSS-treated males and in RGS10^−/−^ females in all experimental groups, this population of monocytes had many cells which expressed reduced levels of CD11b compared to RGS10^+/+^ mice (Fig. 7A). Significant genotype effects were also observed for T-cells. RGS10 deficiency was associated with reductions in circulating CD4^+^ T-cell populations in males and females, with the greatest differences observed in males that did not receive MPTP (Fig. 7B). Male RGS10^−/−^ mice also had reduced CD8^+^ T-cell populations, but no significant genotype differences in these cells were found in females (Fig. 7B).

These few alterations in PBMC populations did not correspond to marked effects of circulating cytokine levels. No significant differences were observed in levels of IL-10, IL-12 p70, IL-1β, IL-2, IL-4, IL-5, IL-6, or CXCL1 in plasma at endpoint. The only differences found were a slight increase in IFNγ in female RGS10^+/+^ H2O-MPTP mice with no effect in males and a decrease in TNF in male RGS10^−/−^ DSS-Saline mice compared to RGS10^+/+^ H2O-Saline mice with no effect in females (Additional file 14).

### ***Colitis and RGS10 deficiency promote sustained CD8***^+^***T-cell-associated immune responses in the brain***

**Fig. 8:**
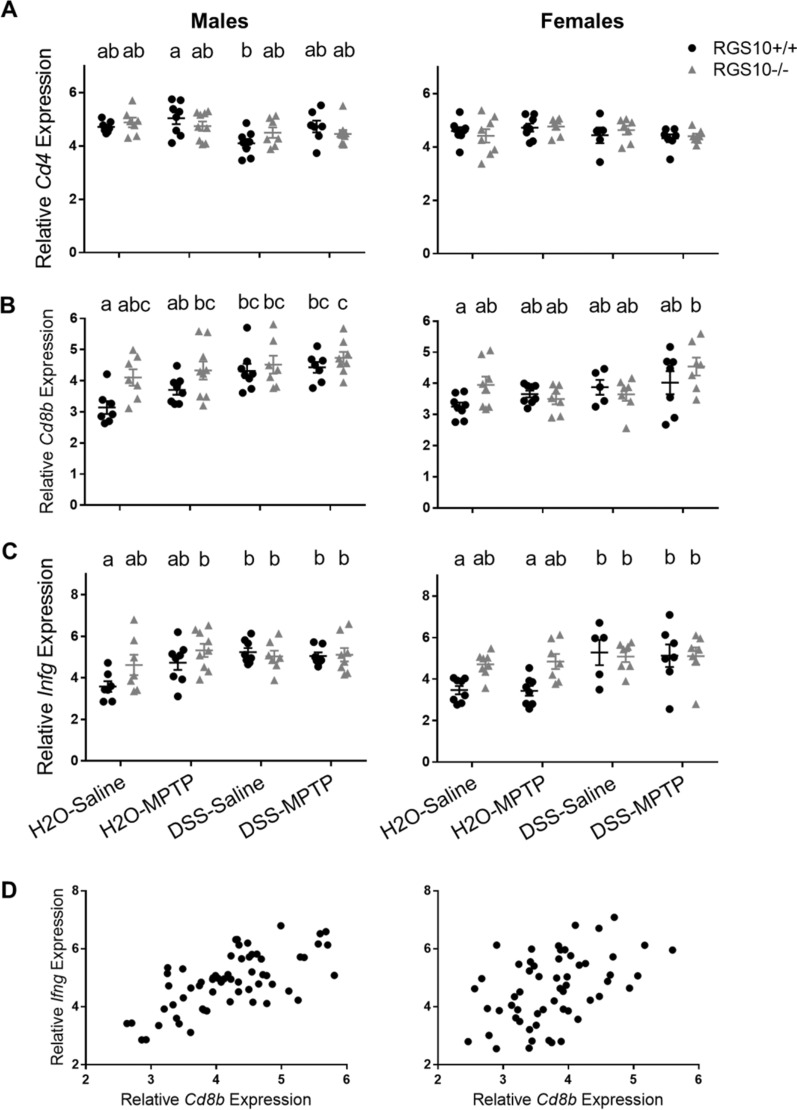
Colitis and RGS10 deficiency induce CD8^+^ T-cell infiltration and elevated Ifng expression in the SNpc. **A** *Cd4*, **B** *Cd8b*, and **C** *Ifng* mRNA levels in the SNpc of mice given H_2_O or 2% DSS followed by saline or MPTP (n = 5–9 per group (groups distinguished by sex, genotype, and treatment), two-way ANOVA, treatment effect *p* < 0.05 for all except *Cd4* for females, genotype effect *p* < 0.05 for *Cd8b* for males and *Ifng* for females, Tukey’s post hoc). Letters above groups indicate post hoc results; no shared letters indicate significant differences between groups. **D** Significant correlation between nigral *Cd8b* and *Ifng* (n = 56–62 per sex, Pearson’s correlation, *p* < 0.001, R^2^ = 0.4445 for males and R^2^ = 0.2057 for females)

To determine if there were indications that colitis-associated immune responses persisted in the brain weeks after DSS exposure, we evaluated mRNA levels of immune-related markers in the SNpc. We found no differences in the abundances of microglia/myeloid-related mRNA transcripts including *H2-Ab, Il1b, Tnf, Il6, and Tlr4,* in astrocyte-associated *Gfap*, in *Nos2,* or in *Snca*. Effects on *Cd4* expression were minimal, with a significant reduction in male RGS10^+/+^ DSS-Saline compared to RGS10^+/+^ H2O-MPTP mice (*p* = 0.0086) and no significant effects in females (Fig. 8A).

Significant main effects of genotype (*p* = 0.0025) and treatment (*p* = 0.0008) were observed for *Cd8b* expression in males, however, and DSS-treated males of both genotypes as well as RGS10^−/−^ H2O-MPTP males had significantly higher levels of *Cd8b* mRNA compared to RGS10^+/+^ H2O-Saline mice. The highest levels were observed in RGS10^−/−^ DSS-MPTP males (Fig. 8B). Expression of *Ifng*, which encodes a key cytokine produced by CD8^+^ T-cells, was also elevated in the SNpc in these same groups of males (Fig. 8C), and it correlated with *Cd8b* mRNA levels (Fig. 8D). A main effect of treatment on *Cd8b* was also found in females (*p* = 0.0125), but *Cd8b* expression was only significantly increased in RGS10^−/−^ DSS-MPTP females compared to RGS10^+/+^ H2O-Saline controls (*p* = 0.0048) (Fig. 8B). DSS treatment did increase expression of *Ifng* in the SNpc in females, however, and a significant effect of genotype (*p* = 0.0197) was also observed (Fig. 8C).

### CD8^+^ T-cell-associated inflammation in the SNpc is associated with striatal TH levels in males but not females

**Fig. 9:**
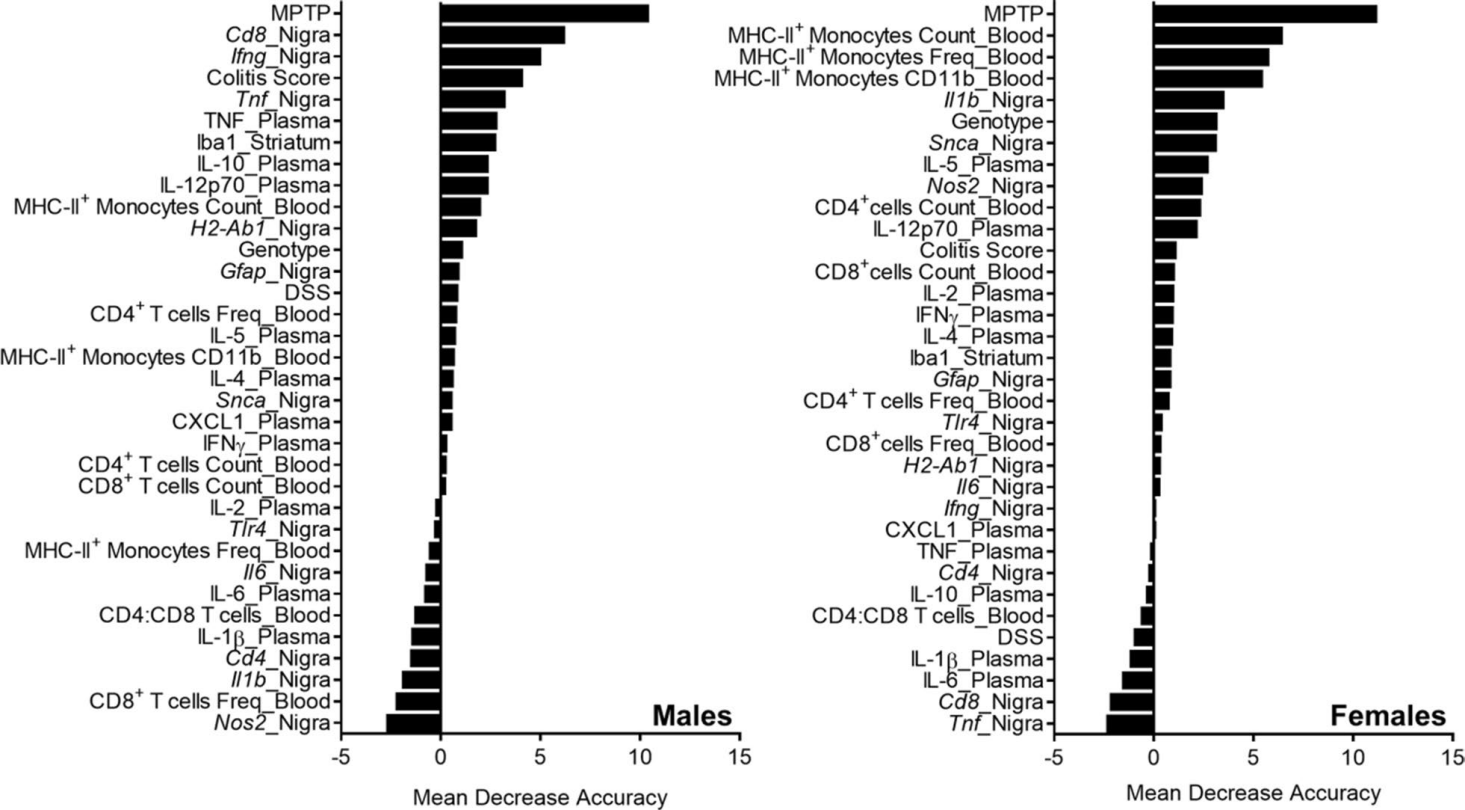
Factors most highly associated with striatal TH levels differ by sex. Ranking of variable importance according to the mean decrease in accuracy of the random forest-based model for males and females when the variable is excluded (target = TH protein in striatum, method = regression, n = 55 per sex, number of trees = 300, number of variables sampled for each tree = 9)

We utilized a random forest algorithm [55] to rank the immune measures obtained in this study according to the closeness of their association with striatal TH levels (Fig. 9). Separate random forests were built for males and females. For both sexes, exposure to MPTP had the strongest impact on levels of striatal TH. In males, *Cd8b* and then *Ifng* expression in the SNpc were the next factors most highly associated with TH followed by colitis DAI score, *Tnf* in the SNpc, and TNF in plasma. After MPTP in females, the most highly ranked factors were counts, frequencies, and the expression of CD11b on MHC-II^+^ monocytes in the blood followed by *Il1b* expression in the SNpc, genotype with respect to RGS10, and *Snca* in the SNpc. Of note, nigral *Cd8b* expression was not found to be useful in building a regression model for striatal TH in females, and colitis score minimally so. Factors that were found to have no association with TH levels in both sexes included concentrations of IL-1β and IL-6 in plasma, the ratio of CD4^+^ to CD8^+^ T-cells in blood, and *Cd4* expression in the SNpc (Fig. 9).

### CD8^+^ T-cell depletion prevents colitis-associated reductions in striatal TH and DAT in males

**Fig. 10:**
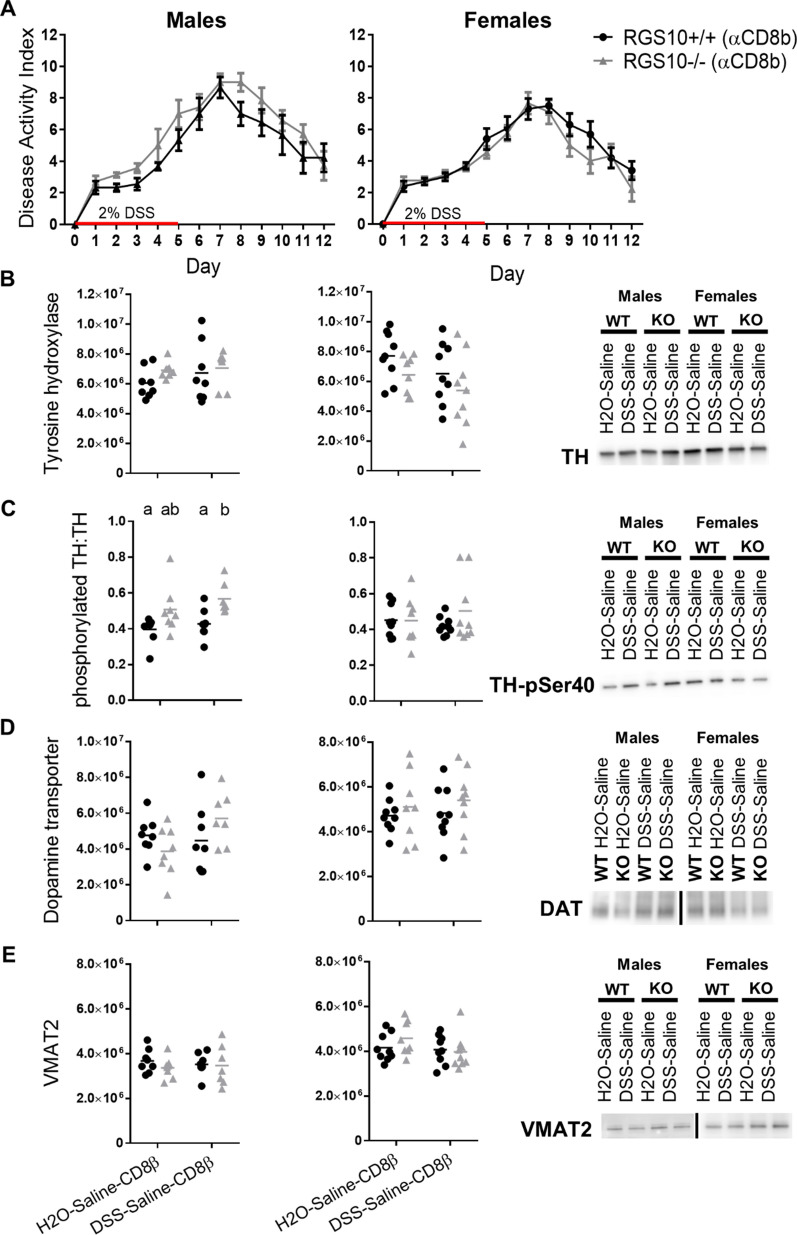
CD8^+^ T-cell depletion prevents DSS colitis-associated reductions in striatal TH and DAT. From CD8^+^ T-cell-depleted mice given H_2_O or DSS followed by saline, **A** disease activity indices of DSS colitis (n = 7–9 per group (groups distinguished by sex, genotype, and treatment); two-way repeated measures ANOVA, Sidak’s post hoc) and **B** TH, **C** ratio of TH phosphorylated at serine 40 to total TH, **D** DAT, and **E** VMAT2 in striatum measured by western blot and normalized to total protein (n = 7–9 per group (groups distinguished by sex, genotype, and treatment); two-way ANOVA, genotype effect *p* = 0.0011 for phosphorylated TH:TH for males, Tukey’s post hoc). Vertical bar indicates blot images are not contiguous. Letter(s) centered above groups reflect results of post hoc tests. Groups that do not share any letter are significantly different from one another

Based on our molecular findings in humans and mice and the predictions of the random forest models, we sought to confirm a direct role of CD8^+^ T-cells in perturbing nigrostriatal activity following colitis. We depleted CD8^+^ T-cells (Additional file 4) and then repeated the regimens of DSS or H2O and then saline injections, producing two new treatment groups: H2O-Saline-CD8b and DSS-Saline-CD8b. In the absence of CD8^+^ T-cells, differences in colitis severity between RGS10^+/+^ and RGS10^−/−^ mice were minimal (Fig. 10A). Importantly, the deficiencies in striatal TH and DAT protein levels relative to controls which were induced by DSS colitis were no longer observed after CD8 depletion (Fig. 10B,D), implicating cytotoxic T-cells as direct mediators of these effects. CD8^+^ T-cell depletion also eliminated the significant increase in the TH-pSer40:TH ratio in DSS-treated RGS10^+/+^ but not RGS10^−/−^ male mice relative to H2O controls (Fig. 10C). There were still no significant reductions in VMAT2 levels with colitis in CD8^+^ T-cell-depleted mice (Fig. 10E). Comparison of striatal protein data from CD8^+^ T-cell-replete and CD8^+^ T-cell-depleted cohorts confirmed significant interactions between CD8^+^ T-cell status and colitis with regard to levels of TH, DAT, and the TH-pSer40:TH ratio but not VMAT2 in male mice (Additional file 15A), indicating that the effect of colitis on these proteins differed significantly when CD8^+^ T cells were missing. In females, significant interactions between CD8^+^ T-cell status and colitis were found with regard to DAT and the TH-pSer40:TH ratio but not TH or VMAT2 (Additional file 15B).

## Discussion

In this study, we sought to confirm and expand upon findings of intestinal inflammation in PD by identifying key peripheral inflammatory mediators that could contribute to neuropathology. We then utilized male and female mouse models to test the potential of gut inflammation, and these inflammatory mediators specifically, to perturb central dopaminergic pathways and/or sensitize them to subsequent neurotoxic insults, providing mechanistic insights into epidemiological connections between IBD and PD and into the theory that, at least in some individuals, PD pathology may originate in the gut. While IBD is a chronic condition, and some animal studies have suggested that chronic but not acute colitis regimens can induce central nervous system (CNS) neurodegeneration [37, 38], this study intentionally employed an acute DSS model in order to assess the persistence of neurological perturbations following self-limited colitis and their potential for augmentation when layered with genetic and chemical risk factors. This study design also controlled the order of effects, ensuring that any exacerbation of MPTP-induced neurological damage in animals that underwent colitis was due to the persistent systemic and CNS effects of colitis and not to concurrent illness.

We observed that expression of inflammation- and immune cell-related genes *LCN2, PTPRC,* and *CD8B* was significantly increased in colon tissue from a small cohort of PD patients compared to matched HCs, as was expression of *SNCA*, which encodes a key protein associated with PD pathology and which is upregulated in the intestine under inflammatory conditions [56]. The increase in *CD8B*, which would be expressed in CD8^+^ T-cells, was of particular interest. In IBD patients, abundant cytotoxic CD8^+^ T-cells are found in affected gut tissue [57], enhanced activation of CD8^+^ T-cells has been documented in peripheral blood [58], and their abundance correlates with measures of IBD-associated inflammation in blood and feces [59, 60]. Furthermore, peripheral blood CD8^+^ T-cells from PD patients exhibit more indicators of activation and fewer indicators of age-related senescence compared to controls [61]. Exposure to α-synuclein peptides stimulates IFNγ production by CD8^+^ T-cells from PD patients [62], and CD8^+^ T-cells have been found in the brains of PD patients *post mortem* [63]. These findings suggest that CD8^+^ T-cells could contribute to ongoing neuroinflammatory immune responses and potentially to nigrostriatal neurodegeneration in PD, and this may be particularly relevant in individuals experiencing heightened cytotoxic T-cell responses related to IBD.

Additionally, we identified markedly increased levels of NFκB p65 in colon tissue from PD patients. While this transcription factor is expressed in different cell types and can mediate different functions in them, and while this study could measure only its expression and not its translocation to the nucleus, its heightened activity in immune cells would explain findings in PD patients of increased levels of NFκB-regulated cytokines and chemokines in colon biopsies [10] and stool [11], and increased fecal calprotectin [7, 8], which can be stimulated through NFκB-associated pathways [64]. Increased TLR4 expression has been identified in the PD gut and contributes to the pathological effects of rotenone in both the gut and brain [9], and TLR4 signaling activates NFκB. It is noteworthy that high NFκB levels were found in all PD patients in this small cohort despite differences in disease duration and treatment and the known heterogeneity of PD manifestations. The consistency of this finding should be evaluated in larger patient populations.

We also discovered that three peripheral blood immune cell types from PD patients had significantly lower levels of an inhibitor of NFκB, RGS10: CD16^+^ and CD14^+^ CD16^+^ monocytes—which had the highest average levels of RGS10—and CD4^+^ T-cells. Heightened NFκB activity in these cells would enhance their activation and promote inflammatory effector functions [12, 17]. Experimentally, excessive NFκB signaling and RGS10 deficiency in myeloid cells have been associated with potentiation of immune-mediated neurotoxicity [15–17, 65]. While it has not been ruled out that the RGS10 deficiency we observed in PD could be due to inherent differences in RGS10 expression in the population which might predispose certain individuals to heightened inflammatory responses, activation of TLR4 signaling cascades has been shown to inhibit RGS10 expression [15, 21], and PD patients reportedly have higher systemic levels of LPS, the classic TLR4 ligand, in their blood [66] along with elevated levels of circulating proinflammatory cytokines [67]. Interestingly, inflammatory bowel disease (IBD) also results in impaired intestinal barrier function and elevated LPS in circulation [68]. These findings suggest another possible mechanism by which intestinal inflammation could potentially increase the risk of PD, through increased NFκB-mediated inflammatory activity in key immune cells enhanced and sustained by systemic downregulation of RGS10 expression.

Whether RGS10 levels in intestinal immune cells are reduced in PD patients as they appear to be in peripheral blood cells remains to be determined and would necessitate isolation of immune cells from larger samples of human gut tissue than were accessible in this study. In fact, the role of RGS10 in the intestine has not been well studied; this research began to address that gap in knowledge by investigating effects of RGS10 loss in the murine gut. Consistent with findings of high RGS10 expression in peripheral myeloid cells in mice [17] and in humans in this study, and with minimal RGS10 expression observed in human gut T-cells [69], we identified RGS10 expression in the mouse colon primarily in cells that expressed the monocyte/macrophage marker CD68. We also observed indicators of intestinal inflammation in RGS10^−/−^ mice. RGS10^−/−^ mice had shorter colons than their WT littermates—a common indicator of inflammation—and higher levels of the transcription factor NFκB p65 and the proinflammatory cytokine IFNγ in colon tissue. While macrophages can produce IFNγ under certain conditions [70], other cell types have been identified as primary contributors to IFNγ responses [71], so the increased levels of this cytokine may reflect the effects of RGS10-deficient macrophages on T-cells and possibly NK cells in the intestinal environment. RGS10^−/−^ mice also developed more severe DSS-induced colitis that resolved more slowly than WT mice. This difference was almost entirely eliminated in mice depleted of CD8^+^ T-cells, indicating that under normal circumstances, crosstalk between RGS10^+^ monocytes/macrophages and cytotoxic T-cells is an important regulator of the severity and resolution of gut inflammation.

Regardless of genotype, CD8-replete male mice exposed to DSS displayed significantly increased expression of the same genes upregulated in PD patient colon biopsies—*Lcn2*, *Ptprc*, *Cd8b*, and *Snca*. Despite similar DAI scores, the inflammatory effects of DSS were somewhat blunted in females, with no significant difference in *Ptprc* and DSS-induced increases in *Cd8b* and *Snca* only in RGS10^−/−^ mice. This is in keeping with reports that female mice are less sensitive to DSS colitis [72], and it may have contributed to limited observation of colitis-related neuropathology in female mice in our study. Our random forest model emphasized that colitis impacted TH^+^ dopaminergic neurons in males much more than female mice but also that the severity of colitis as reflected by the DAI score was a substantially better predictor of striatal TH levels than was the binary classification of DSS or H2O treatment. Our findings are in accordance with the epidemiological studies that found associations between IBD and PD in men but not women [22, 23], and they suggest that sex differences in the degree of inflammation occurring with colitis and/or in the gut-brain crosstalk that occurs with it impact the potential risk for neurological sequelae.

Reductions in levels of TH [73] and DAT [74] are hallmarks of neurological impairment in PD, and these pathological changes can be observed well before the onset of motor symptoms [75]. Reductions in *TH* mRNA in the SNpc have also been reported in PD in association with neurodegeneration [76], and reductions in VMAT2 are associated with presynaptic terminal loss [77]. A key finding in this study is that colitis reduced striatal levels of TH and DAT in males for at least five weeks after the last exposure to DSS. As DSS does not cross the blood–brain barrier, these effects were necessarily mediated by its activity in the periphery [78]. RGS10 deficiency also had a subtle negative impact on dopaminergic neuron health, with TH, DAT, and VMAT2 abundance slightly lower in RGS10^−/−^ mice compared to their RGS10^+/+^ counterparts.

Importantly, we found that colitis alone did not induce significant reductions in VMAT2 in dopaminergic terminals and did not appear sufficient to produce neurodegeneration with dopamine deficiency. While levels of striatal TH decreased in DSS-treated mice, the activity of this enzyme—reflected by the ratio of TH-pSer40:TH—increased in RGS10^−/−^ females and in males of both genotypes. This likely enabled the maintenance of levels of DA and its metabolites. Increased activity of TH has also been observed in brains of PD patients *post mortem* [73]. This compensatory mechanism is not without risk. N-terminal phosphorylation of TH, which increases the enzyme’s activity, also promotes its ubiquitination and degradation [79]. Thus, this modulation can sustain DA levels in the short-term but with time can exacerbate TH deficits, contributing to progressive neurological degeneration such as is observed in PD [80]. These findings indicate that, while acute colitis may not produce substantial neurodegeneration on the time-scale evaluated in this study, its effects may increase the risk for neuron loss over time and/or sensitize an individual to additional neurological insults.

Administration of the neurotoxicant MPTP in rodents has been developed as a model that recapitulates aspects of parkinsonian neurodegeneration, producing striatal TH, DAT, and VMAT2 deficits [81]. The subacute MPTP regimen utilized in this study produced the anticipated results, significantly reducing TH and DAT in the striatum without producing severe neuron damage that may have rendered any augmentation of the phenotype undetectable. The effects of MPTP are typically more severe in male than female mice [82, 83]. While RGS10 loss exacerbated the effects of MPTP in both sexes, significant reductions in VMAT2 were only observed in RGS10^−/−^ males. The effects of colitis appeared to layer with those of MPTP and RGS10 deficiency. Striatal TH levels in male RGS10^−/−^ DSS-MPTP mice were significantly lower than in RGS10^+/+^ or RGS10^−/−^ mice treated with DSS or MPTP alone. Additionally, *Th* gene expression was significantly reduced in the SNpc only in RGS10^−/−^ DSS-MPTP mice, though nigral *Th* appeared to reflect the overall patterns observed in striatal TH.

In investigating which factors were associated with striatal TH levels, we found that in female mice, peripheral blood MHC-II^+^ monocyte measures were the variables most highly ranked. We had also observed that RGS10^−/−^ mice had more MHC-II-expressing Ly-6C^−^ monocytes in peripheral blood and lower CD11b expression on them. A common genetic variant in the human leukocyte antigen (HLA) locus which increases baseline and stimulated expression of MHC-II on peripheral blood monocytes is associated with increased risk for sporadic PD [30, 84]. Infiltration of peripheral monocytes into the CNS has been documented in DSS colitis [85] as well as in models of neurological damage, where these cells are reported to express persistently high levels of MHC-II, to exhibit a proinflammatory phenotype, and to contribute to neurodegeneration [86, 87]. While reduced expression of the integrin CD11b could limit monocyte trafficking, it could also impact the function of these cells in ways which are not currently well-understood [88, 89]. Nigral expression of *Il1b*—which encodes a proinflammatory cytokine primarily produced by myeloid cells—as well as the presence or absence of RGS10—which modulates the inflammatory activity of myeloid cells—were also closely associated with TH levels in females. Though we found no significant differences among experimental groups in many myeloid-related immune markers in the brain, our random forest model suggests that peripheral MHC-II^+^ cells influence dopaminergic neuron health at least in females.

This study identified significant increases in *Cd8b* and *Ifng* expression in the SNpc of DSS-treated males, and in males, these variables were ranked as the most highly associated with striatal TH levels after MPTP. DSS colitis models induce accumulation of CD8^+^ T-cells in the colon, particularly in male mice [72]. Indeed, this study found increased *Cd8b* expression in colon tissue of male mice following acute colitis but more modest changes in females which only reached statistical significance in females lacking RGS10. In mice in which CD8^+^ T-cells had been depleted, DSS colitis did not induce reductions in striatal TH or DAT. The colitis-associated increase in the ratio of phosphorylated to total TH was also mitigated in RGS10^+/+^ but not RGS10^−/−^ male mice. Our findings suggest that CD8^+^ T-cell activity was high in the colons of patients with PD and that these cells were key mediators of neuropathology subsequent to colitis, particularly in male mice, positioning these cells as prime candidates for a mechanistic link between IBD and PD. As this study’s findings were based on mRNA levels and depletion experiments, it will be important to confirm CD8^+^ T-cell localization and degree of infiltration into the brain parenchyma in the nigrostriatal and other brain regions in future studies.

It was also interesting to note that, while no significant differences in nigral *Tnf* expression were found, this measure was one of the most highly ranked in the random forest model for males, as was the concentration of TNF in plasma. Anti-TNF therapy has been reported to significantly reduce the risk for PD development that is otherwise associated with IBD [23], and our experimental findings here as well as other studies [15, 90, 91] support the potential of TNF to promote dopaminergic neuropathology.

## Conclusions

This study provides further evidence that intestinal inflammation is present in the GI tract of individuals with PD and highlights increased levels of *CD8B* and NFκB p65 as important mechanisms which could link GI inflammation with neurodegeneration. It also identifies significant reductions in levels of the NFκB inhibitor RGS10 in immune cells of PD patients and begins to characterize RGS10’s role in regulating neuroimmune mechanisms in the gut-brain axis. This work also demonstrates that intestinal inflammation can perturb and increase the vulnerability of nigrostriatal dopaminergic pathways in WT male mice and that these effects persist after the apparent resolution of gastrointestinal symptoms, lending experimental support for epidemiological studies suggesting that IBD increases the risk for developing PD and highlighting sex dependence of the interaction. CD8^+^ T-cells were found to be critical mediators of colitis-related neuropathology in mice, and given the high level of *CD8B* expression in the colon of PD patients, these cells warrant further investigation as a mechanism linking GI inflammation and the development of PD neuropathology and neurodegeneration in humans.

## Supplementary Information

**Additional file 1**. Characteristics of subjects from whom colon biopsies were collected for evaluation of inflammatory markers.

**Additional file 2**. Criteria for calculation of disease activity index for colitis.

** Additional file 3**. Mouse experiment design. RGS10—regulator of G-protein Signaling 10, DSS—dextran sodium sulfate, MPTP—1-methyl-4-phenyl-1,2,3,6-tetrahydropyridine.

** Additional file 4**. Treatment with anti-CD8β antibody depletes CD8^+^ T-cells from mice. Representative flow cytometry plots confirming **A** acute depletion of CD8^+^ T-cells after two doses of anti-CD8β antibody prior to DSS exposure and **B** sustained depletion of CD8^+^ T-cells at the end of the experiment after weekly doses of anti-CD8β antibody.

**Additional file 5**. Antibodies used in this study. Conjugates are indicated in bold.

**Additional file 6**. Full unedited blots. Annotated, uncropped images of each of the western blots presented in the manuscript as well as β-actin or total protein controls. The portions of the images used in figures are indicated.

**Additional file 7**. qPCR primers used in this study.

**Additional file 8**. Gating strategy for human PBMCs.

**Additional file 9**. Gating strategy for mouse PBMCs.

**Additional file 10**. Characteristics of subjects in which RGS10 levels were evaluated in peripheral blood mononuclear cells. Healthy control (HC) and Parkinson’s Disease (PD) groups compared with two-tailed t-test.

**Additional file 11**. Neurons and lymphoid cells do not express RGS10 in the murine colon at steady state. Myenteric plexus was peeled from fixed colon tissue of RGS10^−/−^ or RGS10^+/+^ mice and probed for RGS10 and PGP9.5. Frozen sections were probed for RGS10 and immune cell markers CD19, CD3, CD4, and CD8 (40 × magnification).

**Additional file 12**. Mice in DSS-Saline and DSS-MPTP groups experience similarly severe colitis, and RGS10 deficiency exacerbates colitis. Disease activity indices of mice assigned to DSS-Saline and DSS-MPTP groups (n = 7–9 per group (groups distinguished by sex, genotype, and treatment); two-way repeated measures ANOVAs, genotype effect *p* = 0.0094 for male DSS-Saline, *p* = 0.0052 for male DSS-MPTP, *p* = 0.0061 for female DSS-Saline, and *p* < 0.0001 for female DSS-MPTP).

**Additional file 13**. Levels of factors regulating dopamine production, packaging, and reuptake are significantly correlated. **A** Relationships between relative mRNA levels encoding tyrosine hydroxylase (TH) in SNpc and TH protein in striatum and among **B** TH, dopamine transporter, and VMAT2 in striatum from male and female mice in all experimental groups (n = 58–63 per sex; Pearson’s correlation).

**Additional file 14.** Minimal impact of genotype or treatment on plasma cytokines. Cytokines in plasma measured at endpoint by multiplexed immunoassay (n = 6–8 per group (groups distinguished by sex, genotype, and treatment), two-way ANOVA, treatment effect *p* = 0.0234 for IFNγ and *p* = 0.0445 for TNF, Tukey’s post hoc). Letter(s) centered above groups reflect results of post hoc tests. Groups that do not share any letter are significantly different from one another.

**Additional file 15.** Significant interactions between CD8^+^ T-cell status and colitis with regard to striatal protein levels. Effects of CD8^+^ T-cell status, colitis, and genotype and the interaction of CD8^+^ T-cell status and colitis on levels of striatal proteins measured by western blot in **A** male and **B** female mice evaluated by generalized linear model.

## Data Availability

The data generated during this study are available from the corresponding author upon reasonable request.

## Abbreviations

PD: Parkinson’s disease
MPTP: 1-Methyl-4-phenyl-1,2,3,6-tetrahydropyridine
RGS10: Regulator of G-Protein Signaling 10
NFκB: Nuclear Factor Kappa B
SNpc: Substantia nigra pars compacta
GI: Gastrointestinal
LPS: Lipopolysaccharide
TLR4: Toll-like Receptor 4
CD: Cluster of differentiation
TNF: Tumor necrosis factor
IFNγ: Interferon gamma
IL: Interleukin
CXCL: C-X-C motif ligand
CRP: C-reactive protein
IBD: Inflammatory bowel disease
DSS: Dextran sodium sulfate
WT: Wild-type
RUMC: Rush University Medical Center
HCs: Healthy controls
PBMCs: Peripheral blood mononuclear cells
AAALAC: Association for Assessment and Accreditation of Laboratory Animal Care
PBS: Phosphate-buffered saline
OCT: Optimal cutting temperature
PFA: Paraformaldehyde
s.c.: Subcutaneously
DAI: Disease activity index
EDTA: Ethylenediaminetetraacetic acid
RT: Room temperature
RIPA: Radioimmunoprecipitation assay
SDS: Sodium dodecylsulfate
dNTPs: Deoxyribonucleotide triphosphates
TBST: Tris-buffered saline with 0.1% Tween-20
HRP: Horseradish peroxidase
qPCR: Quantitative polymerase chain reaction
Ct: Cycle threshold
HPLC: High performance liquid chromatography
PVDF: Polyvinylidene difluoride
DA: Dopamine
DOPAC: 3,4-Dihydroxyphenylacetic acid
HVA: Homovanillic acid
L-DOPA: L-3,4-dihydroxyphenylalanine
MSD: Meso Scale Discovery
FACS: Fluorescence-activated cell sorting
GMFIs: Geometric mean fluorescence intensities
NS: Normal serum
ABC: Avidin–biotin-peroxidase complex
DAB: 3,3′-Diaminobenzidine
NSAIDs: Non-steroidal anti-inflammatory drugs
ANOVA: Analysis of Variance
TH: Tyrosine hydroxylase
DAT: Dopamine transporter
VMAT2: Vesicular monoamine transporter 2
IRB: Institutional Review Board
TH-pSer40: Phosphorylation of tyrosine hydroxylase at serine 40
