# Supplementary material for: Experimental colitis promotes sustained, sex-dependent, T-cell-associated neuroinflammation and parkinsonian neuropathology
Source: Acta Neuropathol Commun. 2021 Aug 19;9:139. doi: 10.1186/s40478-021-01240-4 (PMC8375080; doi:10.1186/s40478-021-01240-4)
Supplement: Supplementary file 2 — Additional file 2. Criteria for calculation of disease activity index for colitis. [file 40478_2021_1240_MOESM2_ESM.pdf]

Score	Weight Loss (%)	Feces Consistency	Fecal blood
0	Gain – 1.99%	firm, dry, well-formed pellets	no blood detected
1	2.0-7.99%	soft, moist, loose pellets	
2	8-13.99%	semi-liquid feces, no rectal adherence	Positive hemoccult test
3	14-19.99%		Visible blood in fecal smear
4	Over 20%	liquid feces, rectal adherence	Visible bleeding from rectum
