# Supplementary material for: Experimental colitis promotes sustained, sex-dependent, T-cell-associated neuroinflammation and parkinsonian neuropathology
Source: Acta Neuropathol Commun. 2021 Aug 19;9:139. doi: 10.1186/s40478-021-01240-4 (PMC8375080; doi:10.1186/s40478-021-01240-4)
Supplement: Supplementary file 3 — Additional file 3. Mouse experiment design. RGS10—regulator of G-protein Signaling 10, DSS—dextran sodium sulfate, MPTP—1-methyl-4-phenyl-1,2,3,6-tetrahydropyridine. [file 40478_2021_1240_MOESM3_ESM.pdf]

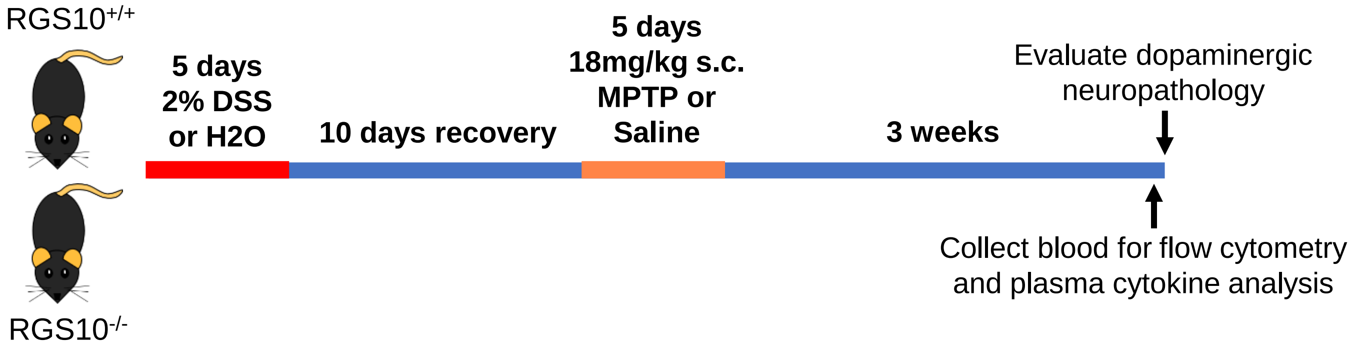
RGS10$^{+/+}$
RGS10$^{-/-}$
5 days
2% DSS
or H2O
10 days recovery
5 days
18mg/kg s.c.
MPTP or
Saline
3 weeks
Evaluate dopaminergic
neuropathology
Collect blood for flow cytometry
and plasma cytokine analysis
