# Supplementary figures and images for: Experimental colitis promotes sustained, sex-dependent, T-cell-associated neuroinflammation and parkinsonian neuropathology
Source: Acta Neuropathol Commun. 2021 Aug 19;9:139. doi: 10.1186/s40478-021-01240-4 (PMC8375080; doi:10.1186/s40478-021-01240-4)

**A**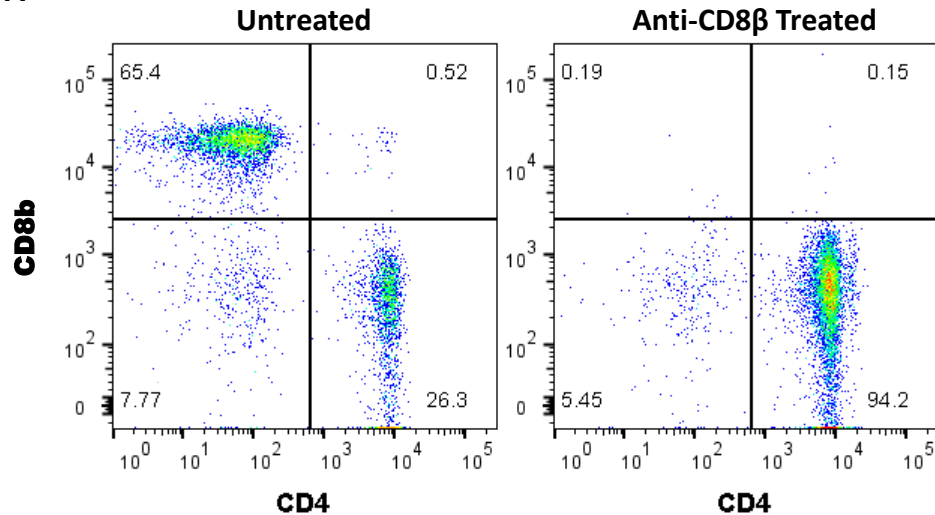**B**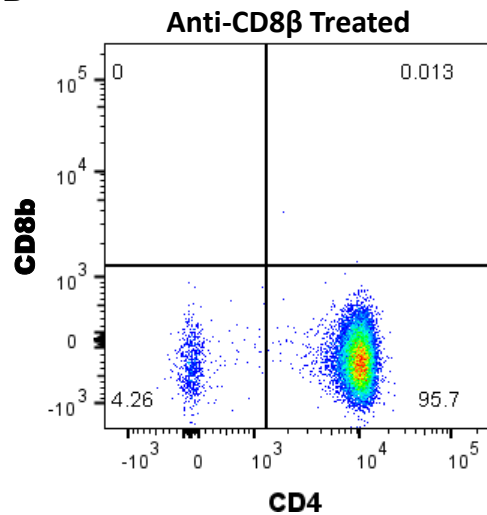

Supplement: Supplementary file 4 — Additional file 4. Treatment with anti-CD8β antibody depletes CD8+ T-cells from mice. Representative flow cytometry plots confirming A acute depletion of CD8+ T-cells after two doses of anti-CD8β antibody prior to DSS exposure and B sustained depletion of CD8+ T-cells at the end of the experiment after weekly doses of anti-CD8β antibody. [file 40478_2021_1240_MOESM4_ESM.pdf]

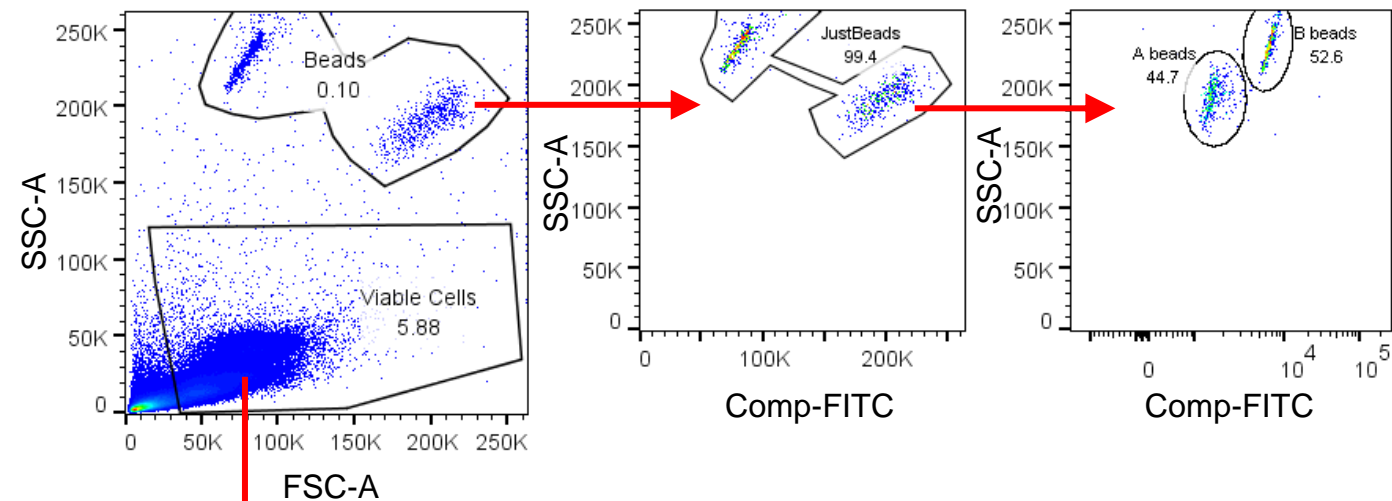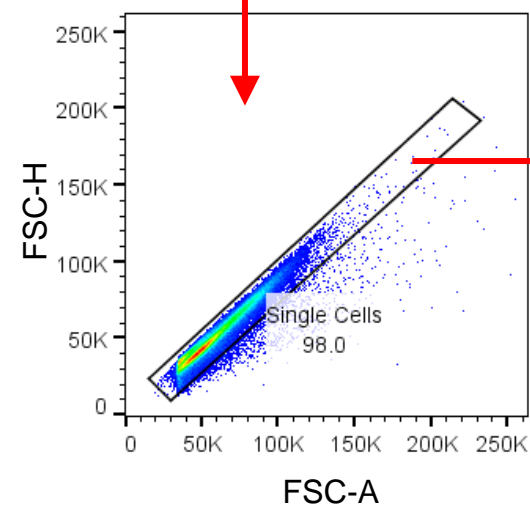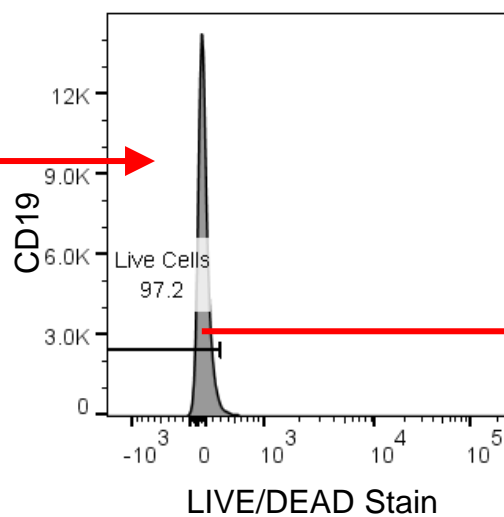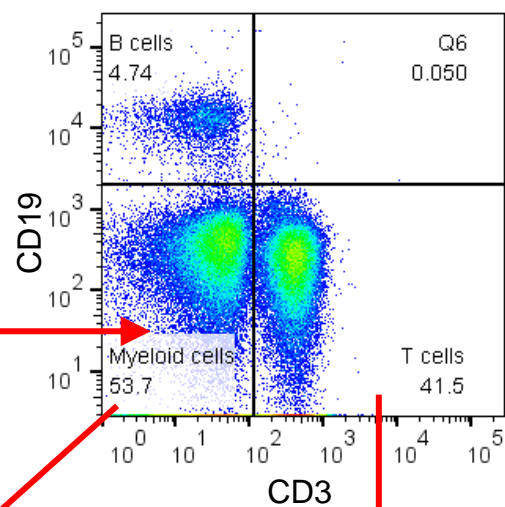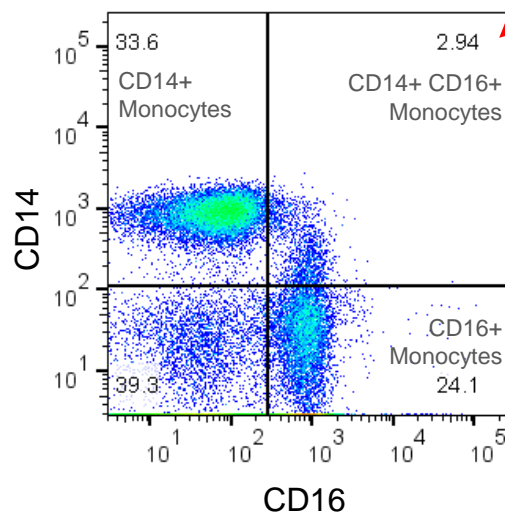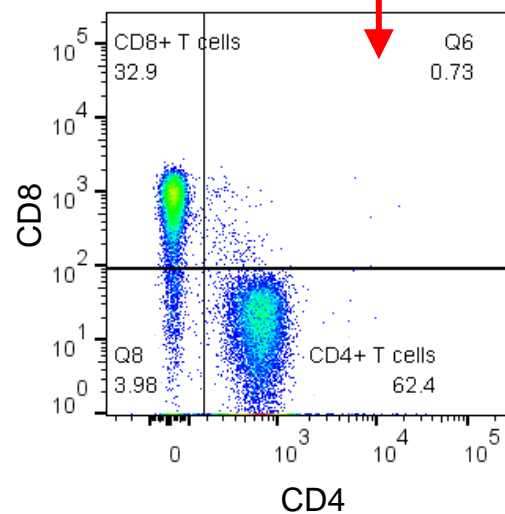

Supplement: Supplementary file 8 — Additional file 8. Gating strategy for human PBMCs. [file 40478_2021_1240_MOESM8_ESM.pdf]

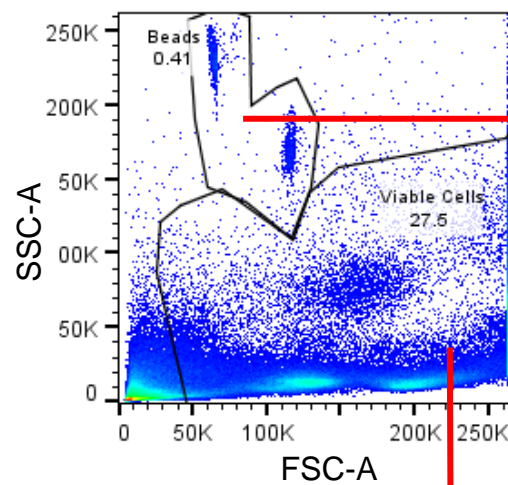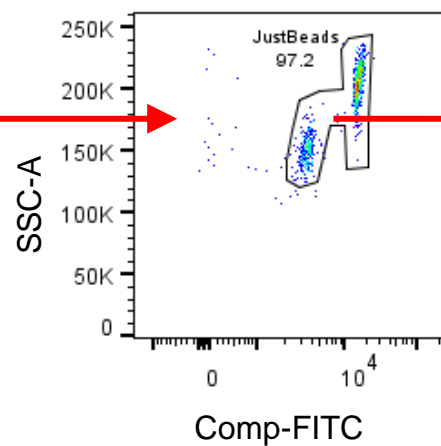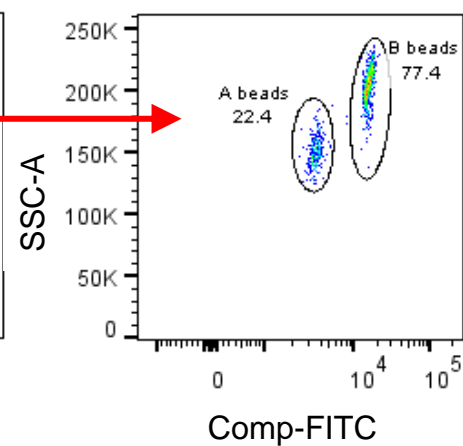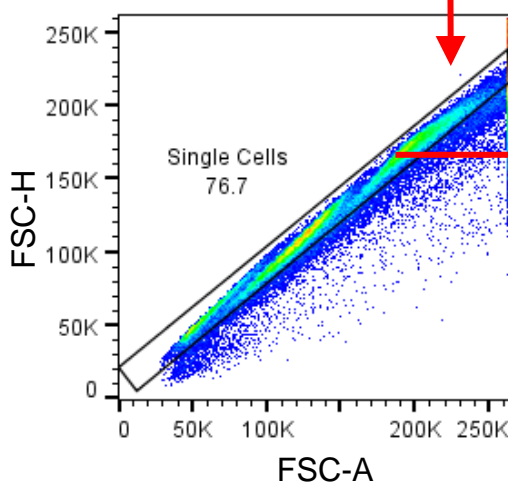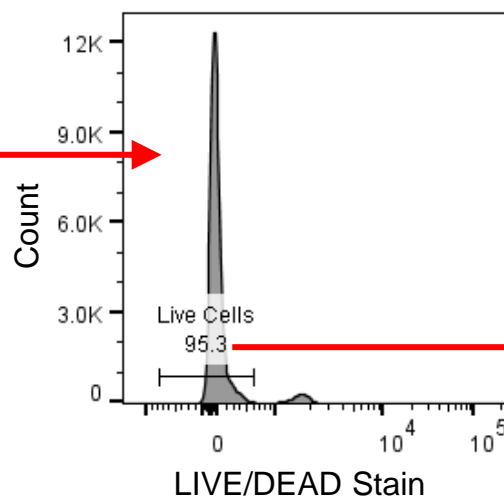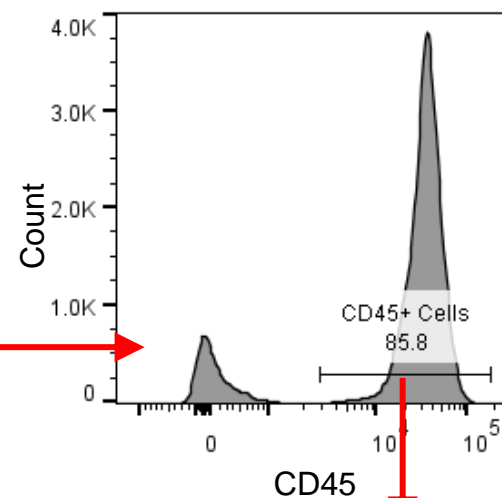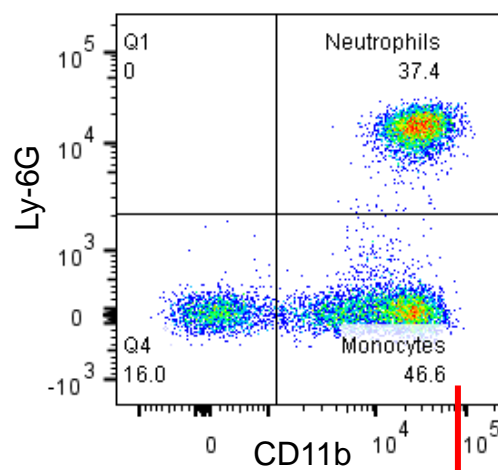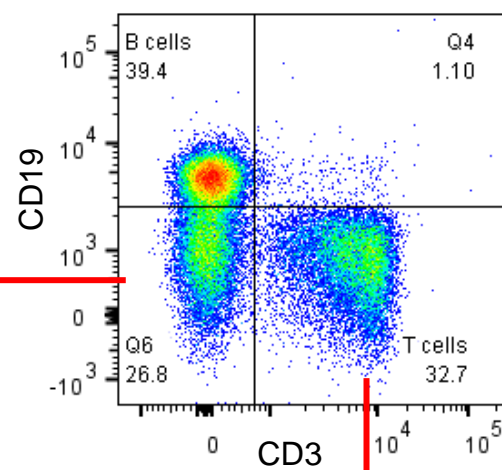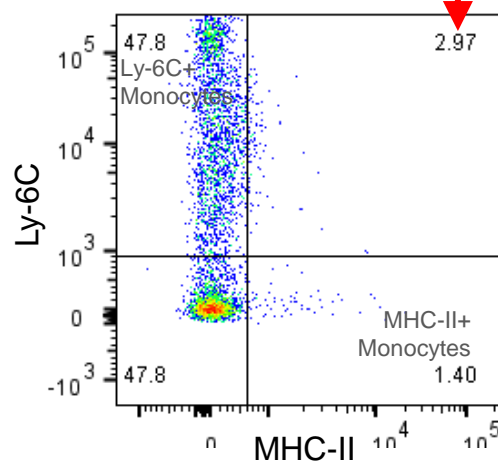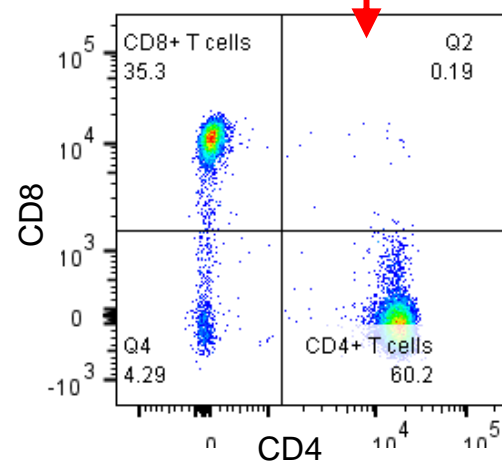

Supplement: Supplementary file 9 — Additional file 9. Gating strategy for mouse PBMCs. [file 40478_2021_1240_MOESM9_ESM.pdf]

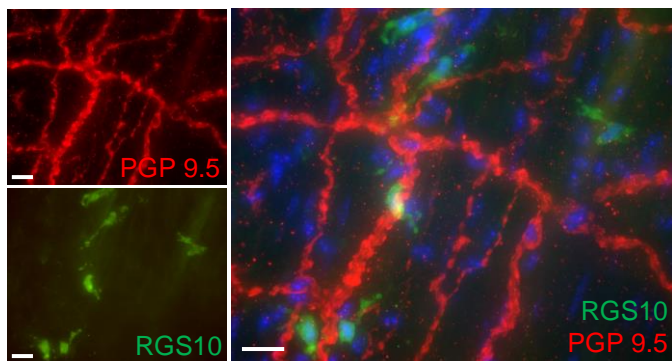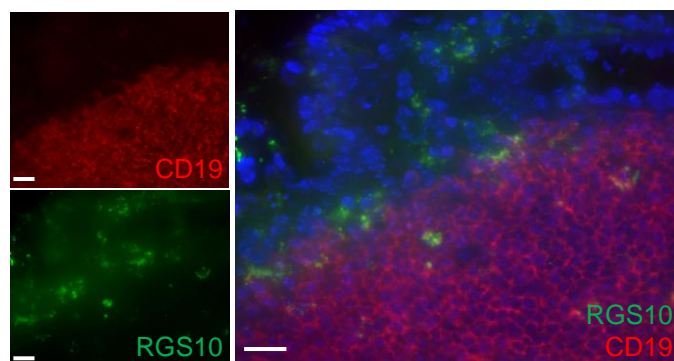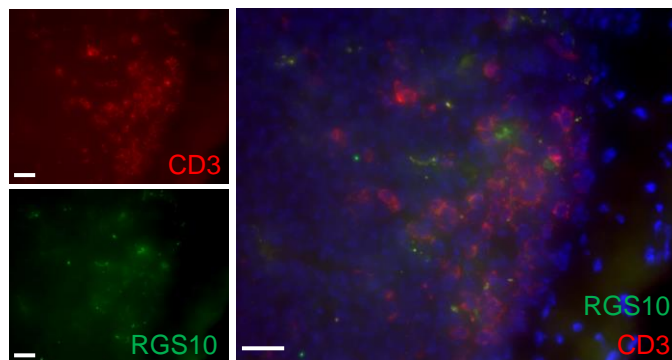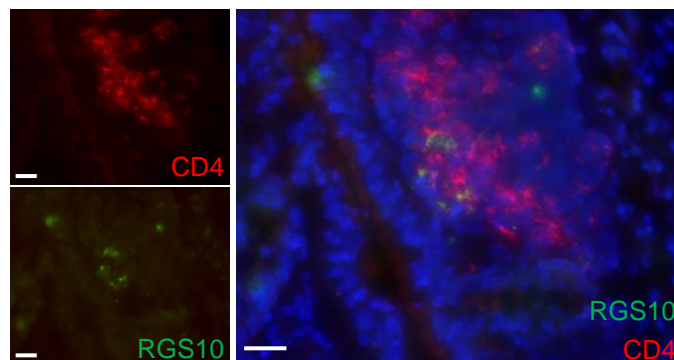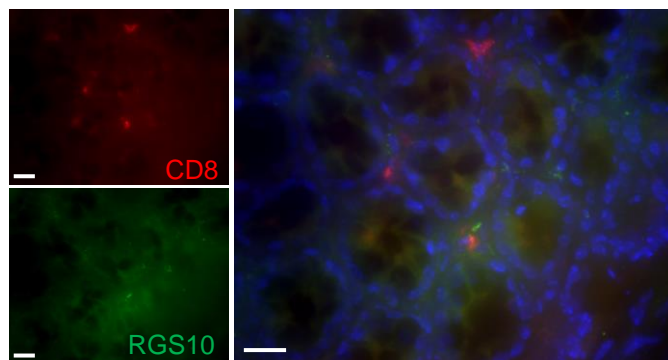

Supplement: Supplementary file 11 — Additional file 11. Neurons and lymphoid cells do not express RGS10 in the murine colon at steady state. Myenteric plexus was peeled from fixed colon tissue of RGS10−/− or RGS10+/+ mice and probed for RGS10 and PGP9.5. Frozen sections were probed for RGS10 and immune cell markers CD19, CD3, CD4, and CD8 (40 × magnification). [file 40478_2021_1240_MOESM11_ESM.pdf]

## Males

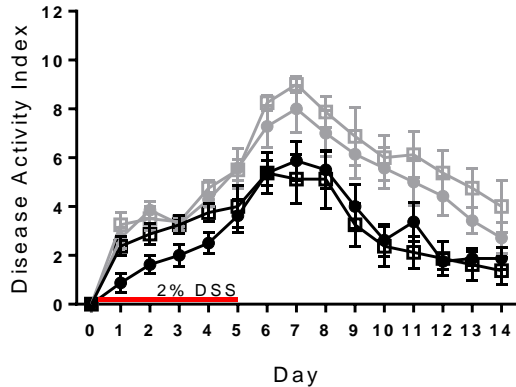

## Females

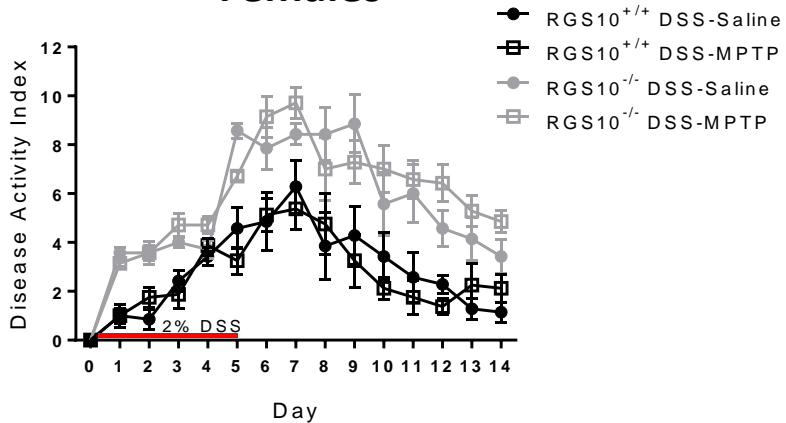

Supplement: Supplementary file 12 — Additional file 12. Mice in DSS-Saline and DSS-MPTP groups experience similarly severe colitis, and RGS10 deficiency exacerbates colitis. Disease activity indices of mice assigned to DSS-Saline and DSS-MPTP groups (n = 7–9 per group (groups distinguished by sex, genotype, and treatment); two-way repeated measures ANOVAs, genotype effect p = 0.0094 for male DSS-Saline, p = 0.0052 for male DSS-MPTP, p = 0.0061 for female DSS-Saline, and p < 0.0001 for female DSS-MPTP). [file 40478_2021_1240_MOESM12_ESM.pdf]

A

Males

Females

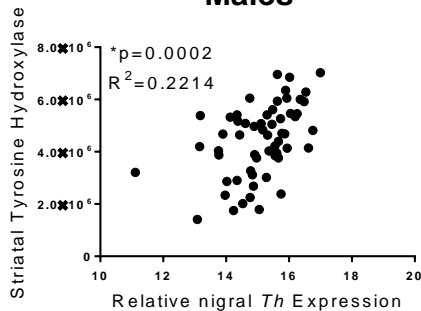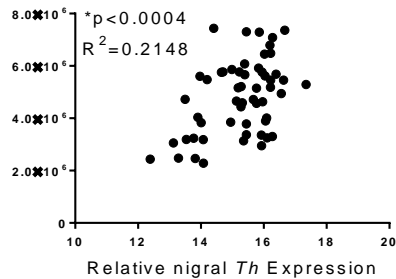

B

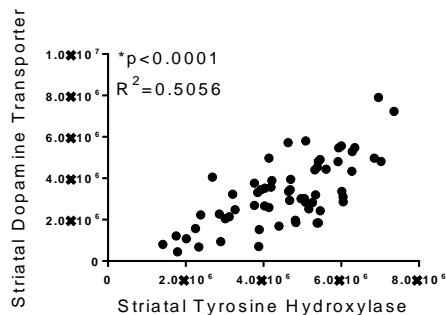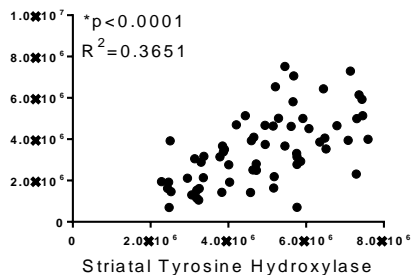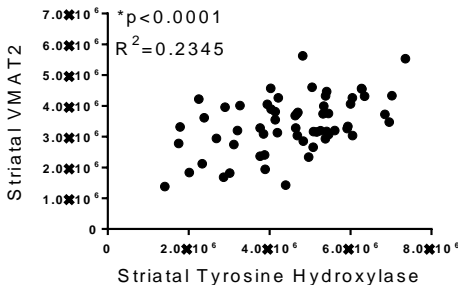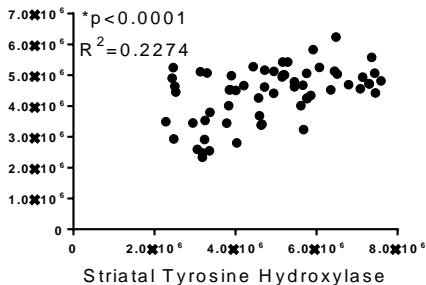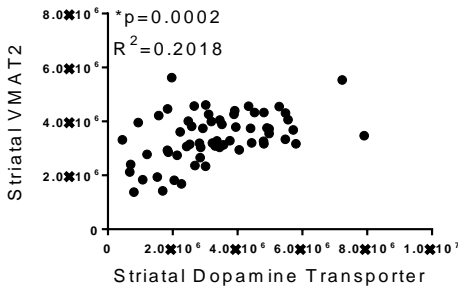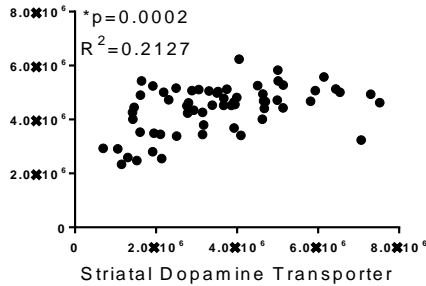

Supplement: Supplementary file 13 — Additional file 13. Levels of factors regulating dopamine production, packaging, and reuptake are significantly correlated. A Relationships between relative mRNA levels encoding tyrosine hydroxylase (TH) in SNpc and TH protein in striatum and among B TH, dopamine transporter, and VMAT2 in striatum from male and female mice in all experimental groups (n = 58–63 per sex; Pearson’s correlation). [file 40478_2021_1240_MOESM13_ESM.pdf]

## Females

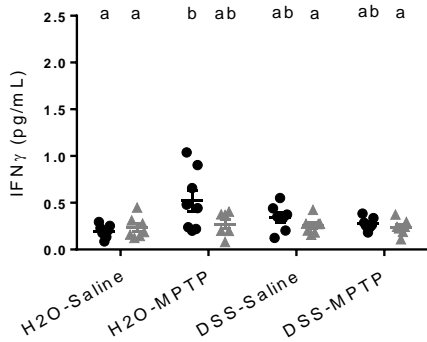

## Males

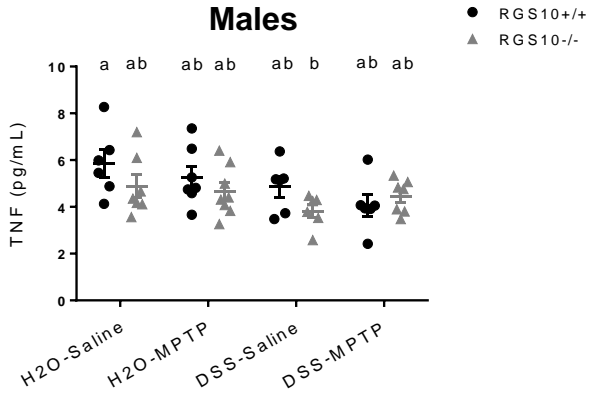

Supplement: Supplementary file 14 — Additional file 14. Minimal impact of genotype or treatment on plasma cytokines. Cytokines in plasma measured at endpoint by multiplexed immunoassay (n = 6–8 per group (groups distinguished by sex, genotype, and treatment), two-way ANOVA, treatment effect p = 0.0234 for IFNγ and p = 0.0445 for TNF, Tukey’s post hoc). Letter(s) centered above groups reflect results of post hoc tests. Groups that do not share any letter are significantly different from one another. [file 40478_2021_1240_MOESM14_ESM.pdf]
