# Supplementary material for: Experimental colitis promotes sustained, sex-dependent, T-cell-associated neuroinflammation and parkinsonian neuropathology
Source: Acta Neuropathol Commun. 2021 Aug 19;9:139. doi: 10.1186/s40478-021-01240-4 (PMC8375080; doi:10.1186/s40478-021-01240-4)
Supplement: Supplementary file 5 — Additional file 5. Antibodies used in this study. Conjugates are indicated in bold. [file 40478_2021_1240_MOESM5_ESM.pdf]

Antibody	Manufacturer	Catalog Number	Dilution/Concentration
Gt anti-RGS10	Santa Cruz	sc-6206	1:500 western<br>1:200 staining<br>1:20 flow cytometry
Ms anti-β-actin	Santa Cruz	sc-47778	1:1000
Rb anti-CD68	Abcam	AB125212	1:200
Rb anti-PGP9.5	Millipore	AB1761-I	1:200
Rt anti-CD19	eBioscience	14-0193-85	1:500
Rt anti-CD3	Biolegend	100202	1:500
Rb anti-CD4	Abcam	AB183685	1:500
Rt anti-CD8β	eBioscience	14-0083-82	1:500
Rb anti-NFκB p65	Santa Cruz	sc-372	1:1500
Rb anti-TH	Millipore	AB152	1:1500 western<br>1:1000 staining
Rt anti-DAT	Millipore	MAB369	1:5000
Rb anti-VMAT2	*Shared by WMC*	Lohr, *et al*. 2014 [42]	1:1000
Gt anti-Iba1	Abcam	AB5076	1:2000
Rt anti CD16/CD32	eBioscience	14-0161-85	1:100
Rb anti-TH phospho-Ser40	PhosphoSolutions	P1580-40	1:1000
Rt anti-Ms CD8β	BioXCell	BE0223	100 μg/dose
Gt anti-Rb-**Biotin**	Vector Laboratories	BA-1000	1:500
Gt anti-Ms IgG-**HRP**	BioLegend	405306	1:2000
Gt anti-Rb IgG-**HRP**	Jackson ImmunoResearch	111-035-144	1:2000
Gt anti-Rt IgG-**HRP**	Jackson ImmunoResearch	112-035-006	1:2000
Dk anti-Gt IgG-**AF488**	ThermoFisher Scientific	A-11055	1:500 whole mounts<br>1:1000 frozen sections
Dk anti-Rb IgG-**AF594**	ThermoFisher Scientific	A-21207	1:500 whole mounts<br>1:1000 frozen sections
Dk anti-Rt IgG-**AF594**	ThermoFisher Scientific	A-21209	1:1000
Dk anti-Gt IgG-**PerCP-Cy5.5**	Santa Cruz	sc-45102	1:50
Rt anti-Ms Ly-6G-**PacBlue**	BioLegend	127611	1:100
Rt anti-Ms CD11b-**PE-Cy7**	BioLegend	101215	1:200
Rt anti-Ms CD4-**PE**	eBioscience	12-0041-81	1:100
Rt anti-Ms CD8β-**APC-eFluor780**	eBioscience	47-0083-82	1:100
Rt anti-Ms CD45-**PerCP-Cy5.5**	eBioscience	45-0451-80	1:100
Ah anti-Ms CD3-**PE-610**	eBioscience	61-0031	1:100
Rt anti-Ms MHC-II-**APC**	Miltenyi Biotec	130-102-139	1:50
Rt anti-Ms Ly-6C-**AF488**	eBioscience	53-5932-82	1:200
Rt anti-Ms CD19-**BV650**	BioLegend	115541	1:100
Ms anti-Hu CD14-**PE**	Biolegend	301806	1:100
Ms anti-Hu CD19-**PE-Cy7**	Biolegend	302216	1:20
Ms anti-Hu HLA-DQ-**APC**	eBioscience	17-9881-41	1:100
Ms anti-Hu CD8α-**APC-H7**	BD Biosciences	560273	1:100
Ms anti-Hu CD3-**eFluor450**	eBioscience	48-0037-42	1:100
Ms anti-Hu HLA-DR-**V500**	BD Biosciences	561224	1:20
Ms anti-Hu CD4-**BV650**	Biolegend	317436	1:100
Ms anti-Hu CD16-**AF700**	Biolegend	302026	1:20

Conjugates are indicated in bold. AF-Alexa Fluor, Ah-Armenian hamster, BV-Brilliant Violet, Ch-chicken, DAT-dopamine transporter, Dk-donkey, Gt-goat, Ms-mouse, Hu-human, PGP-protein gene product, Rb-rabbit, Rt-rat, TH-tyrosine hydroxylase, VMAT2-vesicular monoamine transporter 2
