# Supplementary material for: Experimental colitis promotes sustained, sex-dependent, T-cell-associated neuroinflammation and parkinsonian neuropathology
Source: Acta Neuropathol Commun. 2021 Aug 19;9:139. doi: 10.1186/s40478-021-01240-4 (PMC8375080; doi:10.1186/s40478-021-01240-4)
Supplement: Supplementary file 6 — Additional file 6. Full unedited blots. Annotated, uncropped images of each of the western blots presented in the manuscript as well as β-actin or total protein controls. The portions of the images used in figures are indicated. [file 40478_2021_1240_MOESM6_ESM.pdf]

Full, unedited blots for Fig 1A

Red box indicates portion of blot shown in manuscript.

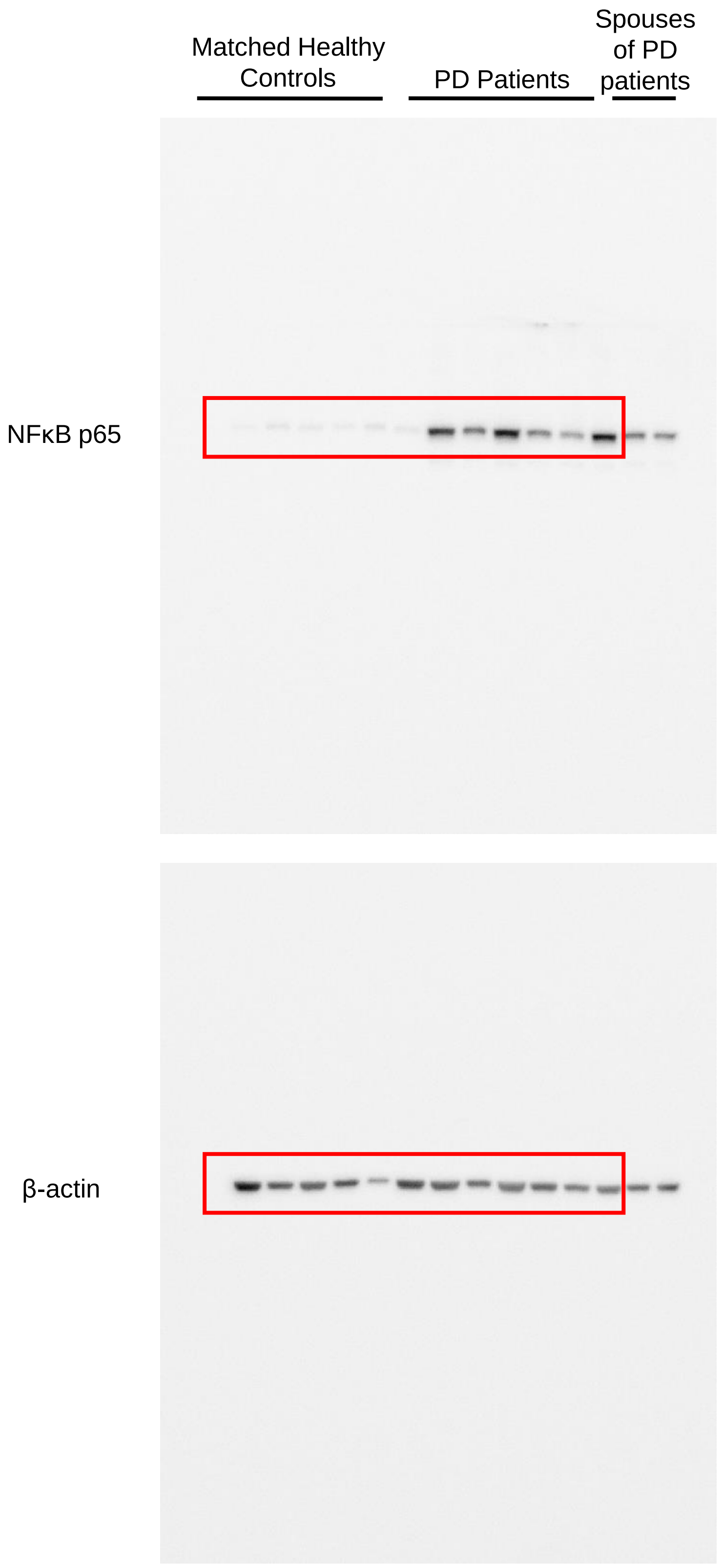

Full unedited blots for Fig 3A

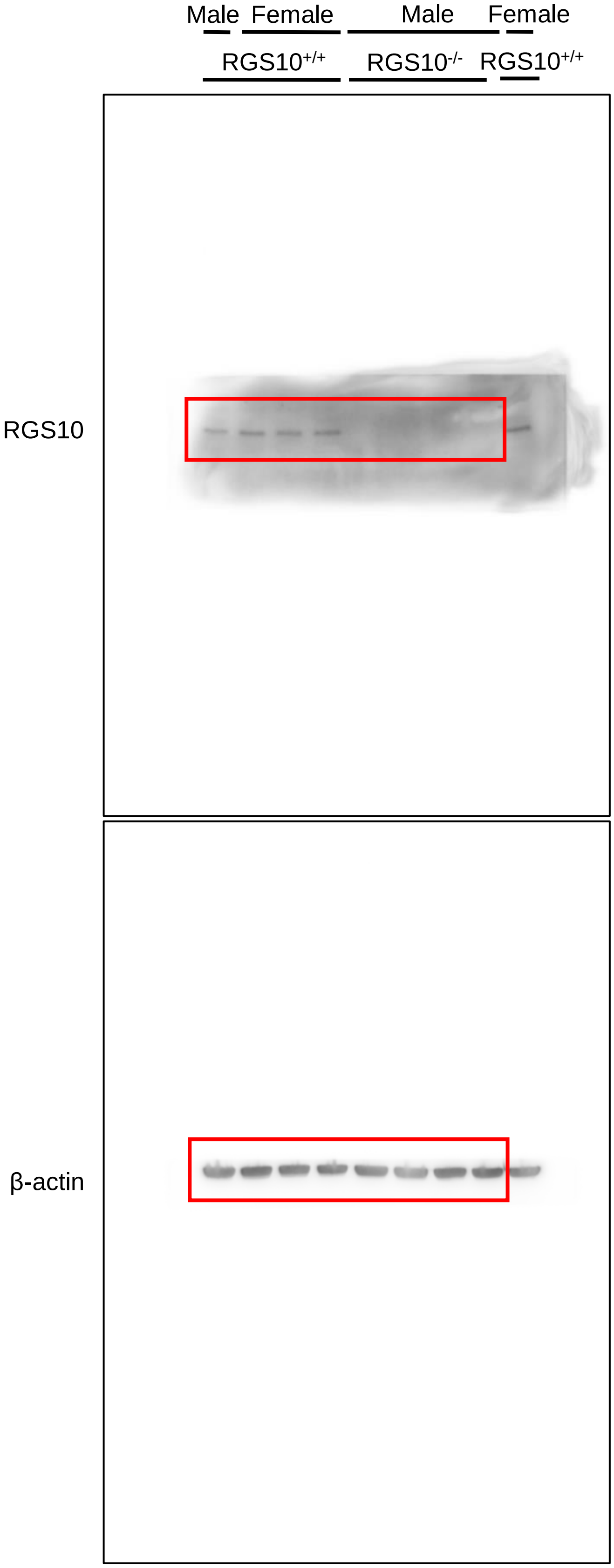

Full unedited blots for Fig 3B

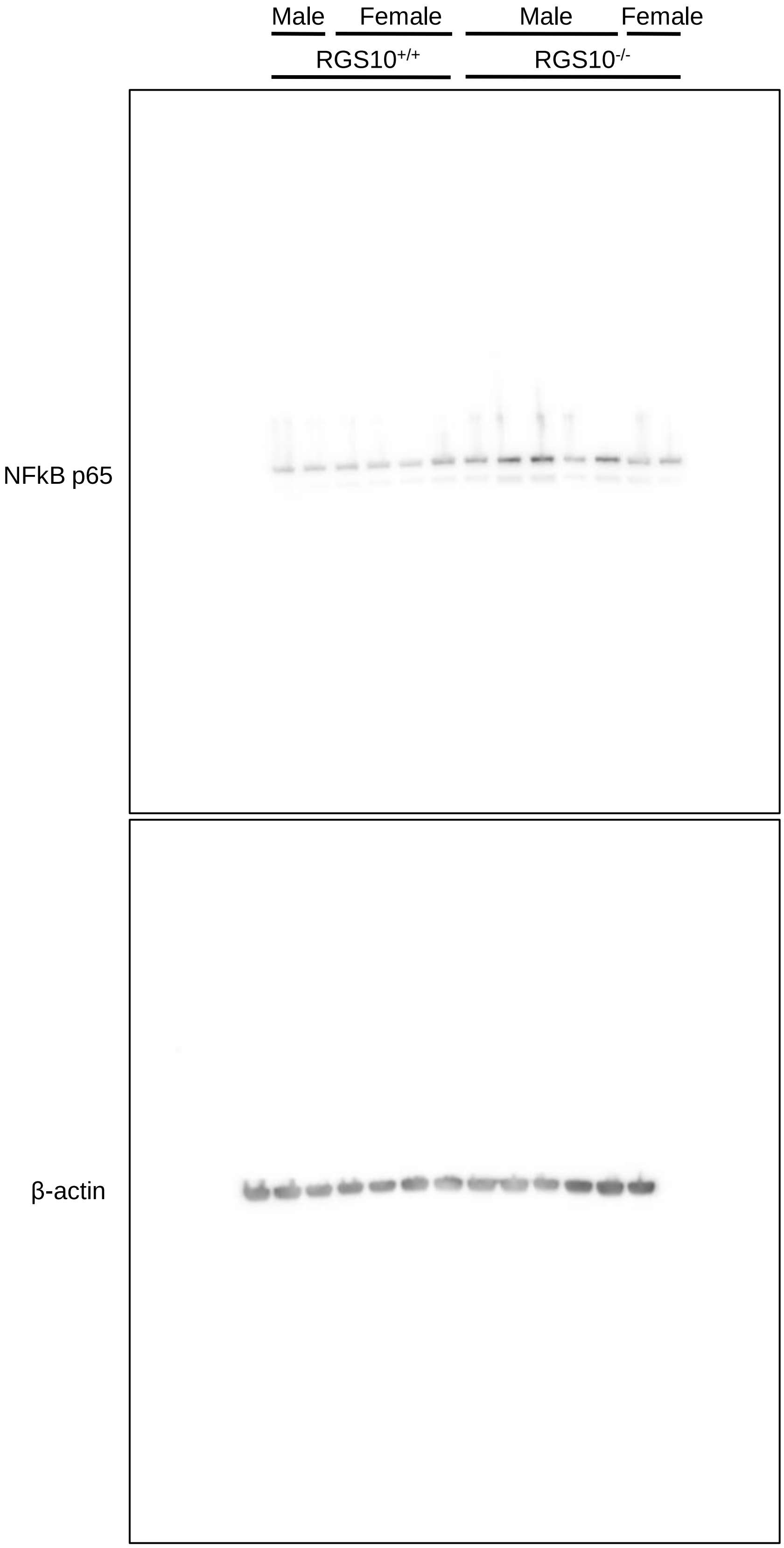

Full unedited blots for Fig 5C

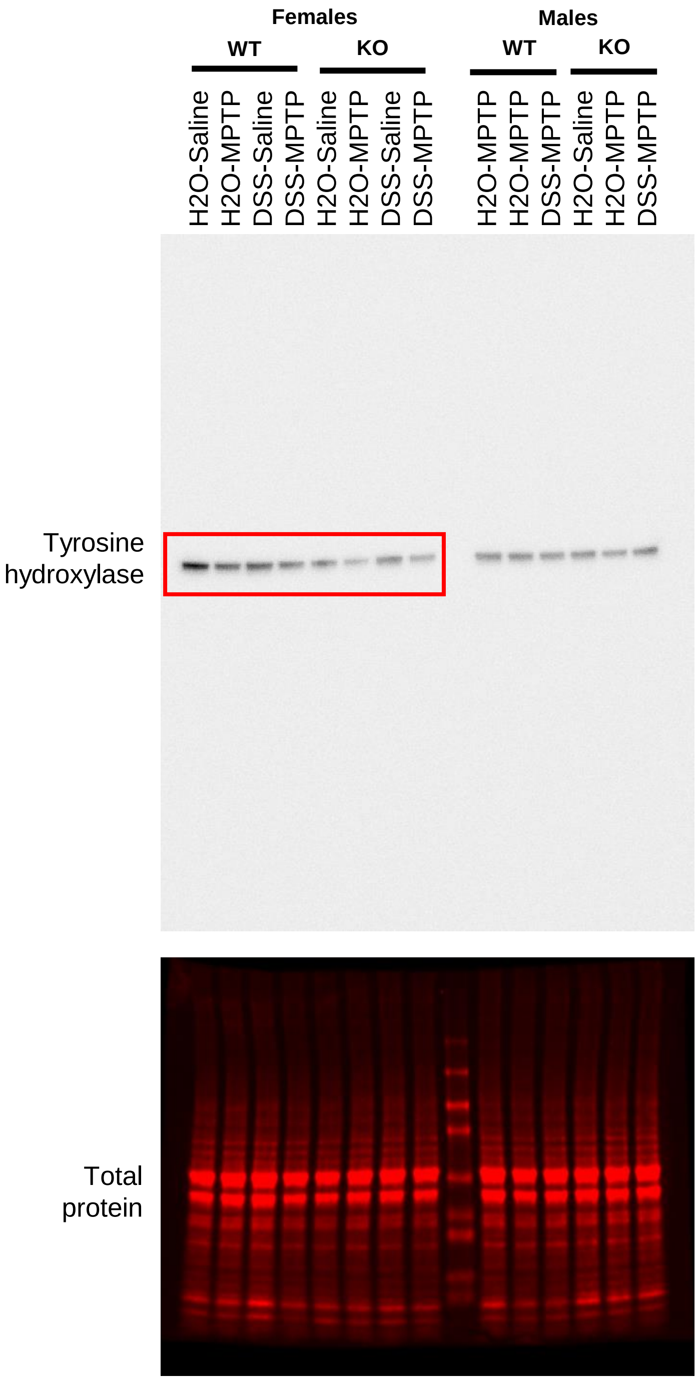

Full unedited blots for Fig 5D

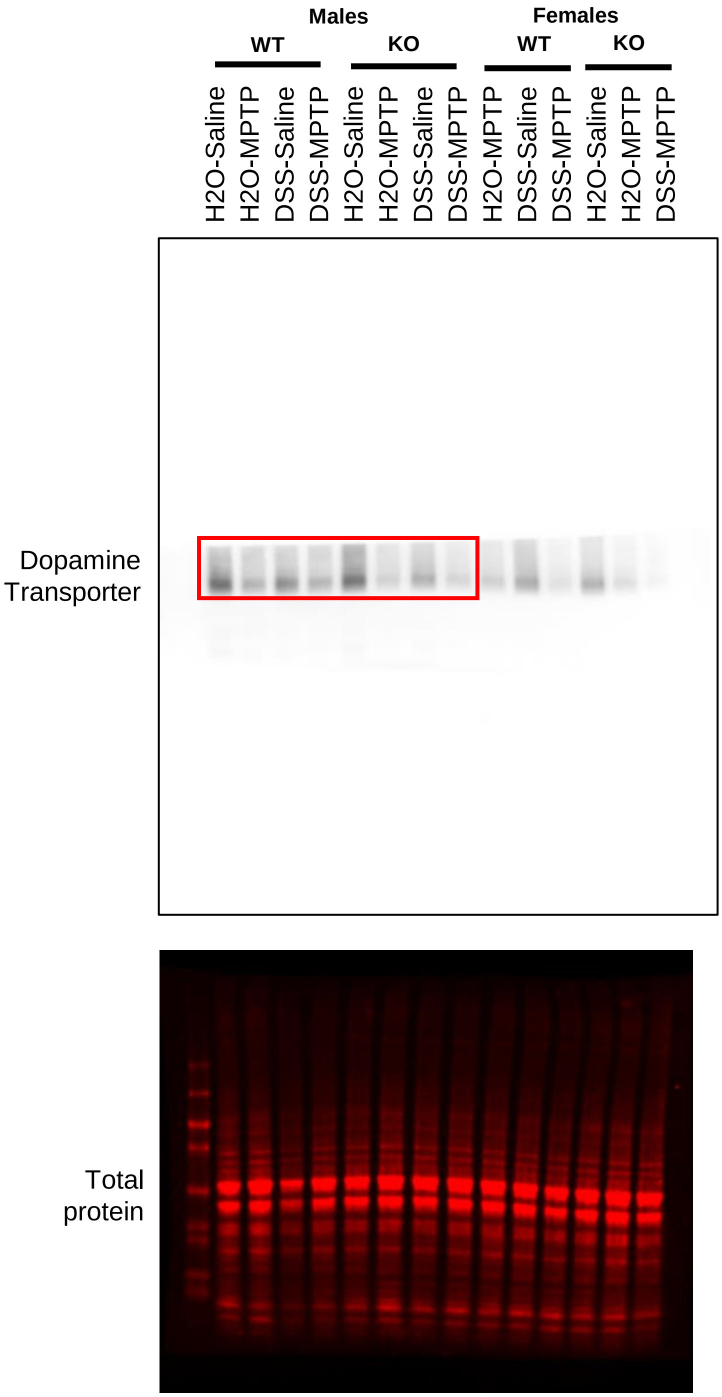

Full unedited blots for Fig 5E

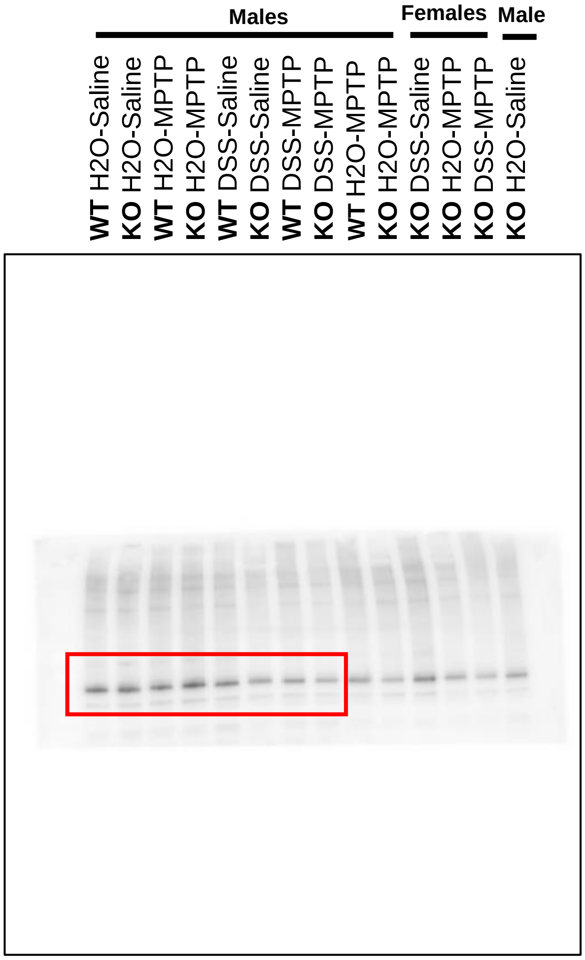

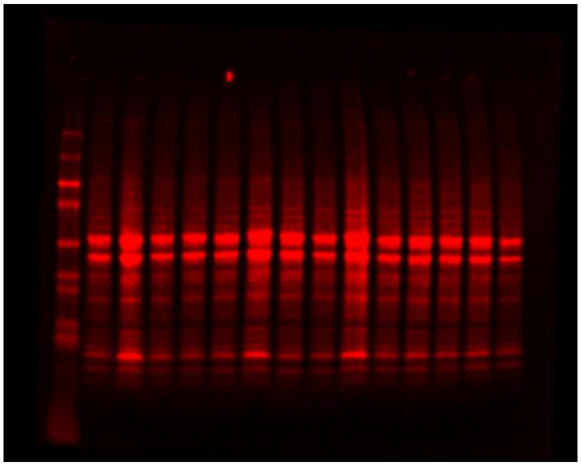

Full unedited blots for Fig 6A

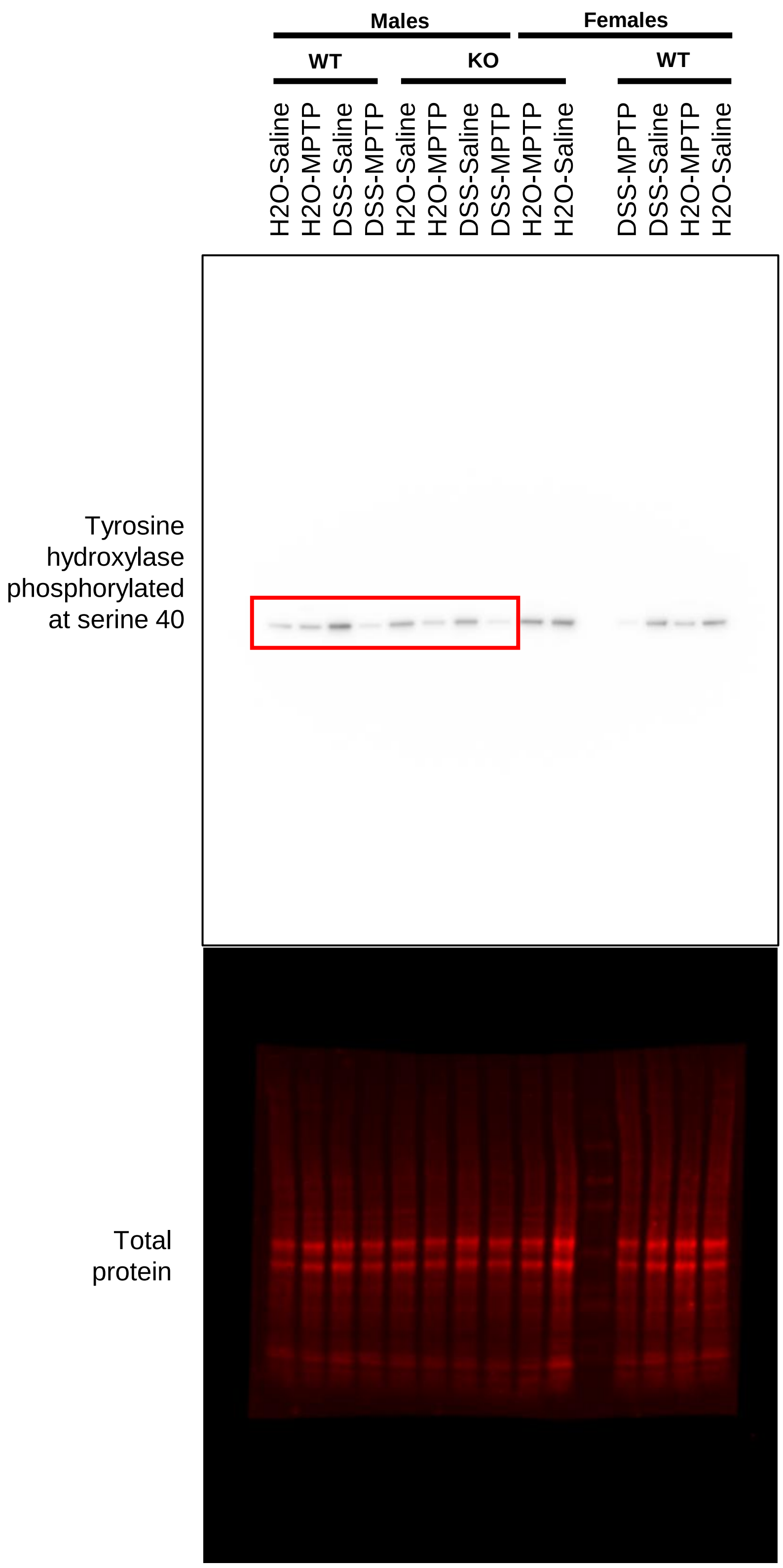

Full unedited blots for Fig 10B

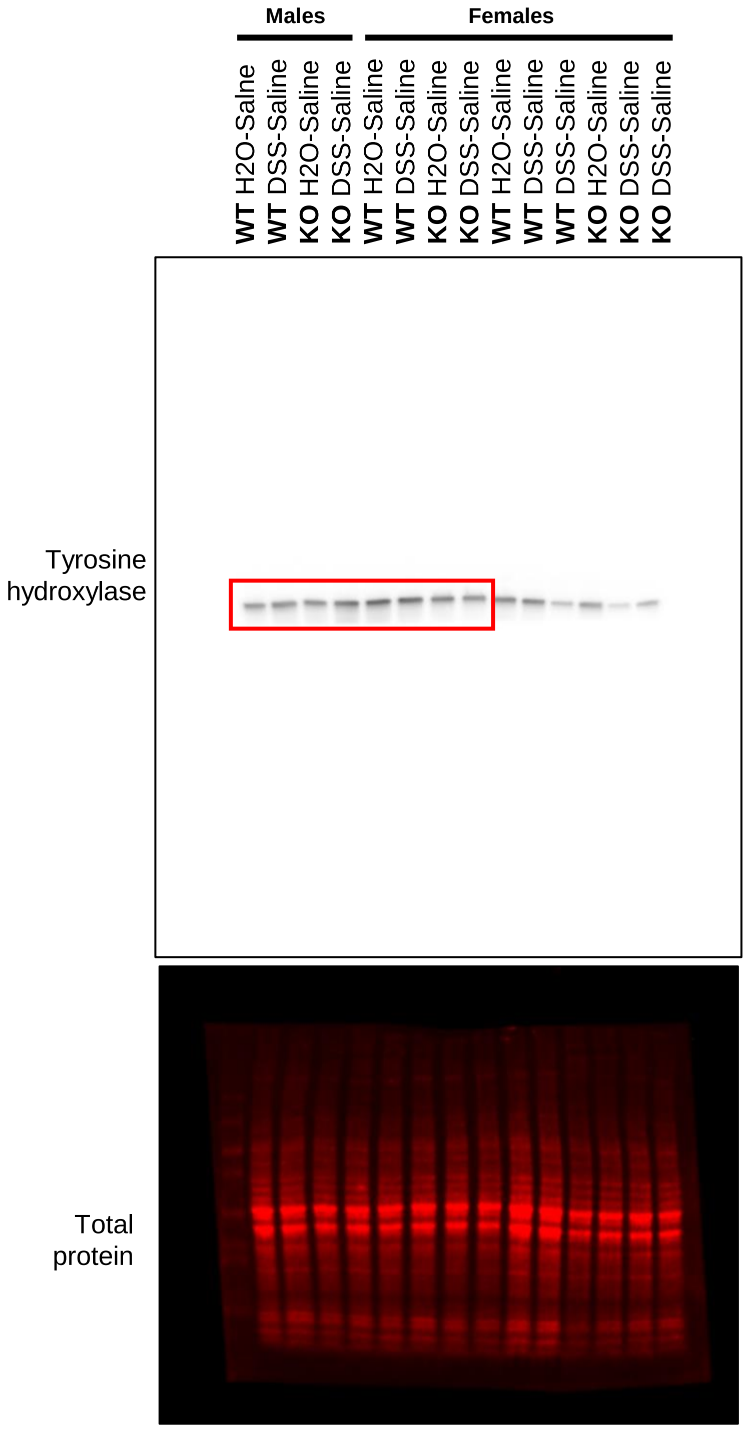

Full unedited blots for Fig 10C

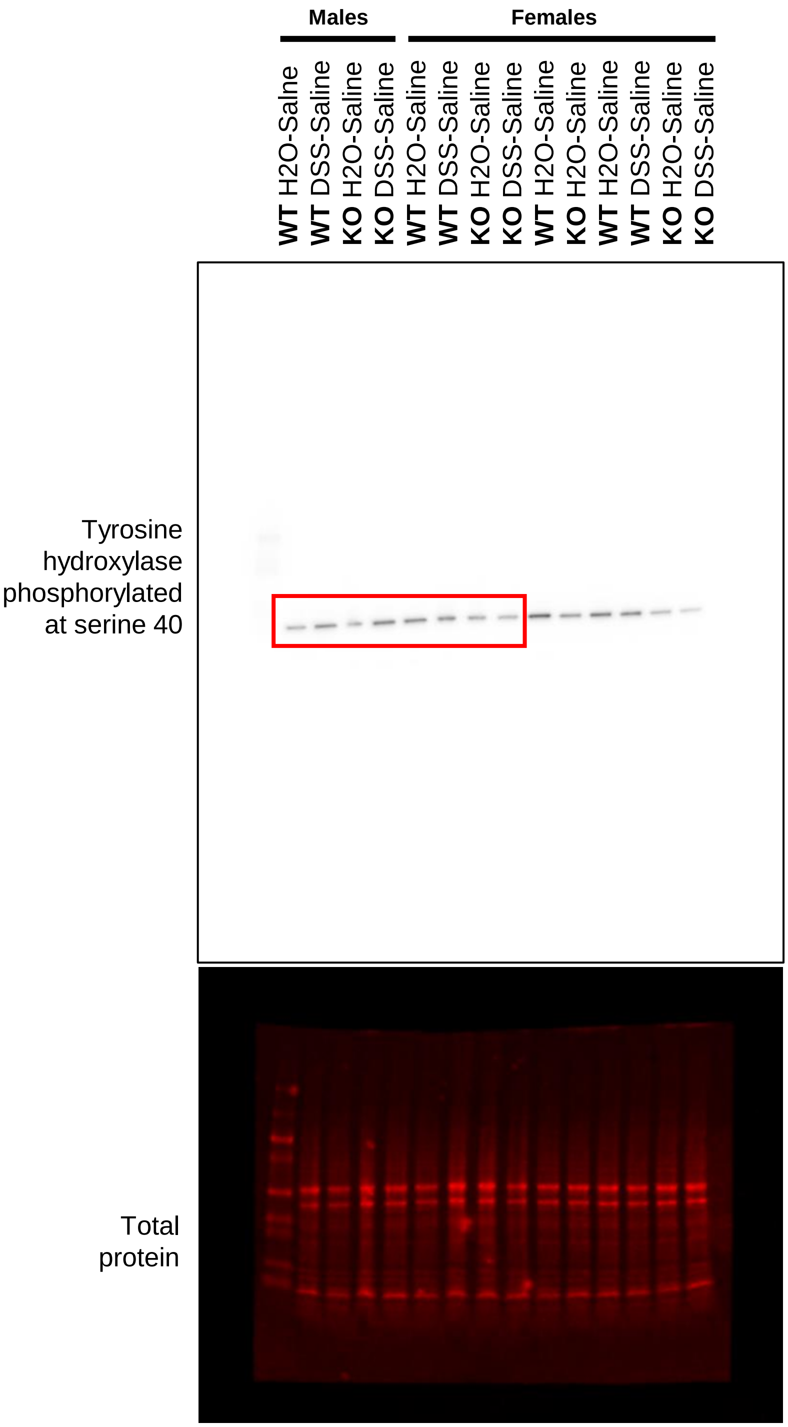

Full unedited blots for Fig 10D

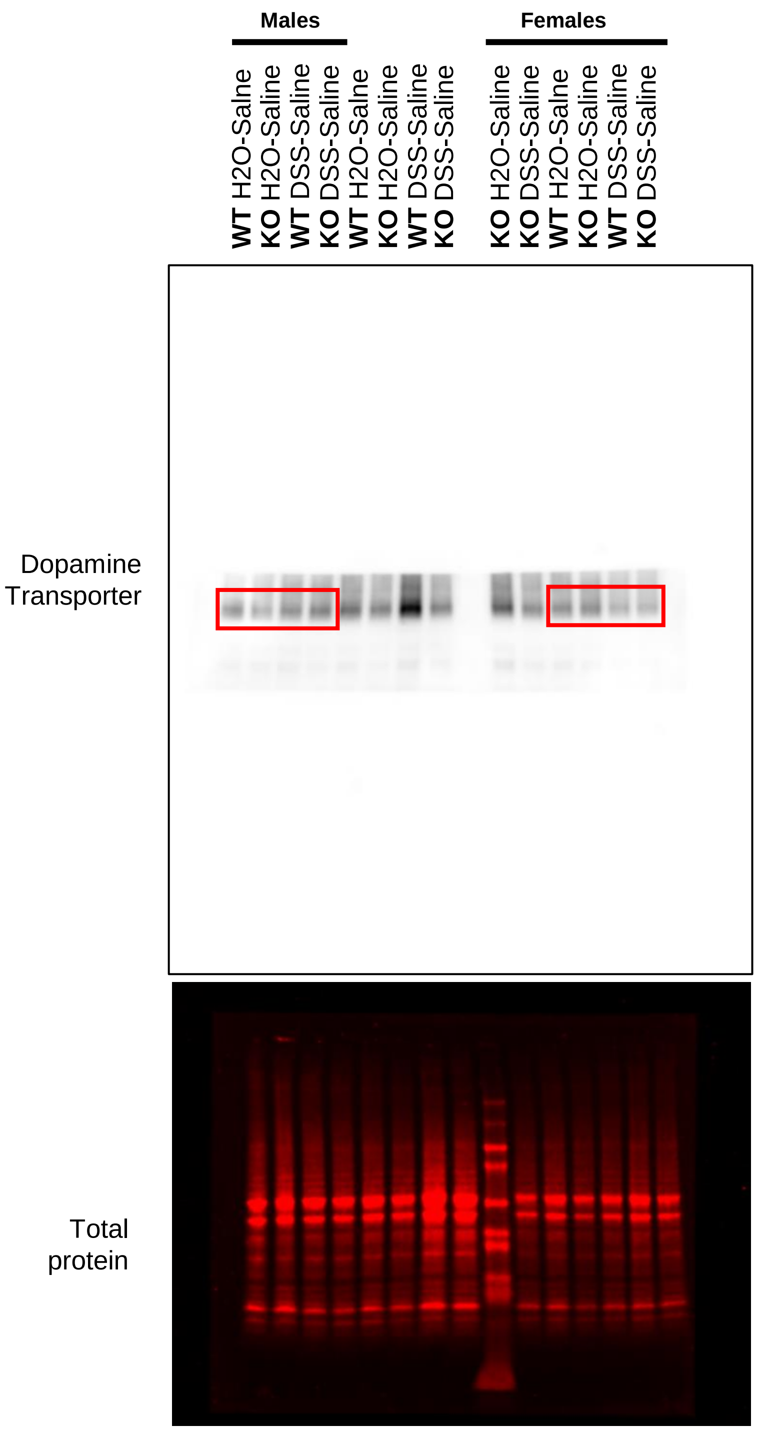

Full unedited blots for Fig 10E

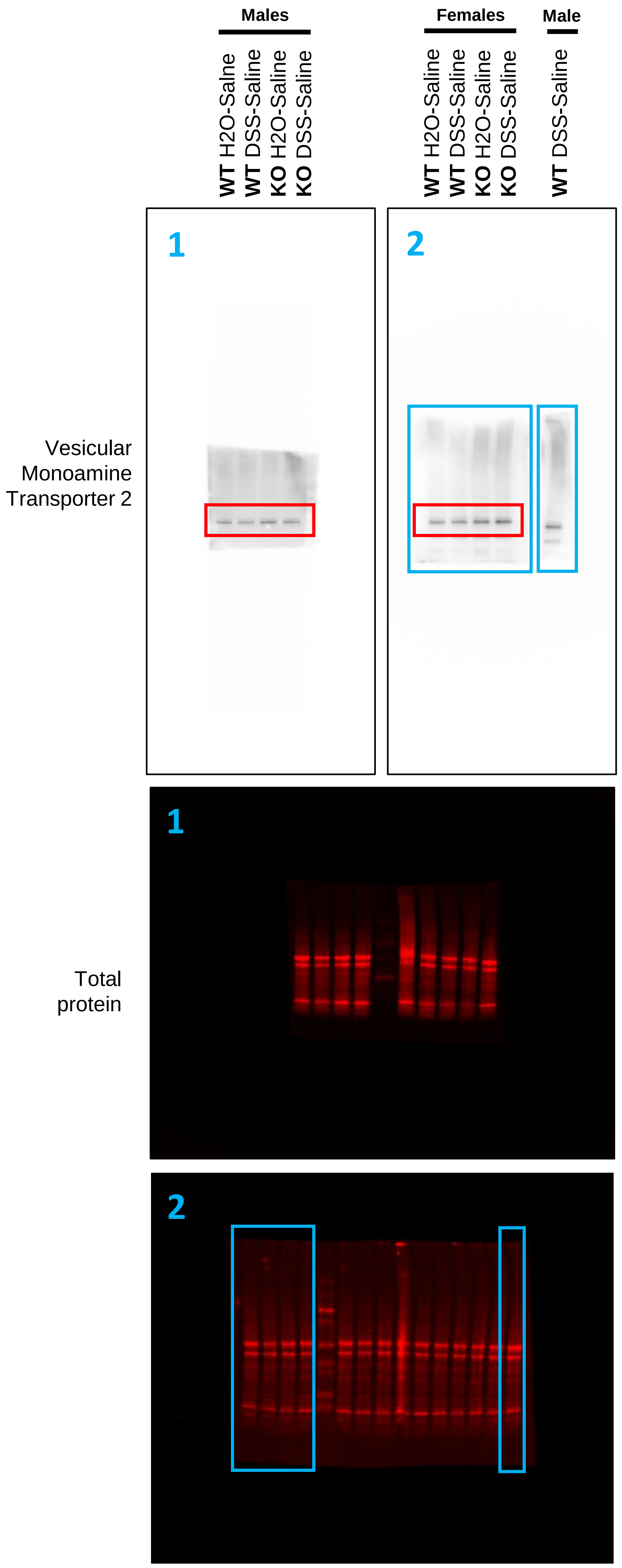

Blue box indicates position of cut blot sections on whole total protein-stained blot, where applicable.
