# Supplementary material for: Experimental colitis promotes sustained, sex-dependent, T-cell-associated neuroinflammation and parkinsonian neuropathology
Source: Acta Neuropathol Commun. 2021 Aug 19;9:139. doi: 10.1186/s40478-021-01240-4 (PMC8375080; doi:10.1186/s40478-021-01240-4)
Supplement: Supplementary file 7 — Additional file 7. qPCR primers used in this study. [file 40478_2021_1240_MOESM7_ESM.pdf]

Gene	Forward Sequence (5'→3')	Reverse Sequence (5'→3')
RNA18SN5	GTAACCCGTTGAACCCCATT	CCATCCAATCGGTAGTAGCG
PPIA	TGCCATCGCCAAGGAGTAG	TGCACAGACGGTCACTCAAA
Gapdh	CAAGGTCATCCATGACAACTTTG	GGCCATCCACAGTCTTCTGG
Ppia	TGGAGAGCACCAAGACAGACA	TGCCGGAGTCGACAATGAT
Chat	AAGGTCGGGTGGACAACATC	GTTTCTCAGAAGCCAGCACG
LCN2	TCACCCTCTACGGGAGAACC	CAGGGAGGCCCAGAGATTTG
Lcn2	TGGAAGAACCAAGGAGCTGT	GGTGGGGACAGAGAAGATGA
NOS2	CCTCCCCGAGGATCCCTCCC	CCTCCCCGCACTCCCTTGTG
Nos2	CAGGAGGAGAGAGATCCGATTTA	GCATTAGCATGGAAGCAAAGA
PTPRC	CATCACAGCGAACACCTCAGATGC	GCGCTTCCAGAAGGGCTCAGA
Ptprc	TCATGGTCACACGATGTGAAGA	AGCCCGAGTGCCTTCCT
CD8B	GACAGTGGCATCTACTTCTG	AAGGAAATCAACCACACTCA
Cd8b	GCTGTCCTTGATCATCACTCTCA	ACTAGCGGCCTGGGACATT
SNCA	CAGGAAGGAATTCTGGAAGAT	TAGTCTTGATACCCTTCCTCA
Snca	AAATGTTGGAGGAGCAGTGG	GAAGGCATTTCATAAGCCTCA
Th	TTGGCTGACCGCACATTT	GCCCCCAGAGATGCAAGT
Cd4	GTGAGCTGGAGAACAGGAAAGAG	GGCTGGTACCCGGACTGA
Ifng	CAAGTTTGAGGTCAACAACC	TCTTATTGGGACAATCTCTTCC
H2-Ab1	CAGGAGTCAGAAAGGACCTC	AGTCTGAGACAGTCAACTGAG
Tnf	CTGAGGTCAATCTGCCCAAGTAC	CTTCACAGAGCAATGACTCCAAAG
Il1b	ATCTTTGAAGAAGAGCCCAT	CCTGTAGTGCAGTTGTCTAA
Il6	GAGGATACCACTCCCAACAGACC	AAGTGCATCATCGTTGTTCATACA
Gfap	TGCTGGAGGGCGAAGAAA	CGGATCTGGAGGTTGGAGAA
Tlr4	ACTGTTCTTCTCCTGCCTGACA	TGATCCATGCATTGGTAGGTAATA
