# Supplementary material for: Experimental colitis promotes sustained, sex-dependent, T-cell-associated neuroinflammation and parkinsonian neuropathology
Source: Acta Neuropathol Commun. 2021 Aug 19;9:139. doi: 10.1186/s40478-021-01240-4 (PMC8375080; doi:10.1186/s40478-021-01240-4)
Supplement: Supplementary file 10 — Additional file 10. Characteristics of subjects in which RGS10 levels were evaluated in peripheral blood mononuclear cells. Healthy control (HC) and Parkinson’s Disease (PD) groups compared with two-tailed t-test. [file 40478_2021_1240_MOESM10_ESM.pdf]

	HC (N=13)	PD (N=33)	Total (N=46)	p value
**Age**				0.260
- Mean (SD)	65.3 (9.9)	69.0 (9.1)	67.9 (9.4)	
- Min - Max	52.0 - 82.0	40.0 - 87.0	40.0 - 87.0	
**Gender**				0.067
- Female	10 (76.9%)	9 (27.3%)	19 (41.3%)	
- Male	3 (23.1%)	24 (72.7%)	27 (58.7%)	
**Caffeine (g/yr)**				0.729
- Mean (SD)	4141.6 (3232.5)	3743.5 (4013.8)	3856.0 (3778.3)	
- Min - Max	533.5 – 11394.6	0.0 – 18082.1	0.0 – 18082.1	
**Tobacco (pack-yrs)**				0.647
- Mean (SD)	4.9 (9.1)	6.5 (14.8)	6.1 (13.3)	
- Min - Max	0.0 - 30.0	0.0 - 75.0	0.0 - 75.0	
**NSAID use (g/yr)**				0.422
- Mean (SD)	380.3 (304.8)	701.2 (2218.2)	610.5 (1882.8)	
- Min - Max	0.0 – 884.0	0.0 – 12480.0	0.0 – 12480.0	

SD-standard deviation, yr-year
