# Supplementary material for: Experimental colitis promotes sustained, sex-dependent, T-cell-associated neuroinflammation and parkinsonian neuropathology
Source: Acta Neuropathol Commun. 2021 Aug 19;9:139. doi: 10.1186/s40478-021-01240-4 (PMC8375080; doi:10.1186/s40478-021-01240-4)
Supplement: Supplementary file 15 — Additional file 15. Significant interactions between CD8+ T-cell status and colitis with regard to striatal protein levels. Effects of CD8+ T-cell status, colitis, and genotype and the interaction of CD8+ T-cell status and colitis on levels of striatal proteins measured by western blot in A male and B female mice evaluated by generalized linear model. [file 40478_2021_1240_MOESM15_ESM.pdf]

A.

Males	Variable	term	estimate	std.error	t.value	p.value	p.value.sig
	TH	CD8_Status_Replete	-1.86E+06	4.14E+05	-4.482925	0.0000369	*
		Colitis_None	-3.92E+05	4.08E+05	-0.9614709	0.3404487	
		Genotype_WT	7.45E+04	2.91E+05	0.2560349	0.7988621	
		CD8_Status:Colitis	1.37E+06	5.81E+05	2.3523349	0.0221963	*
	pTH_TH	CD8_Status_Replete	2.52E-01	4.63E-02	5.4468692	0.0000013	*
		Colitis_None	-4.49E-02	4.48E-02	-1.0026455	0.3205038	
		Genotype_WT	-1.27E-01	3.24E-02	-3.9232142	0.000249	*
		CD8_Status:Colitis	-2.16E-01	6.50E-02	-3.332602	0.0015583	*
	DAT	CD8_Status_Replete	-1.15E+06	5.08E+05	-2.2732411	0.0269391	*
		Colitis_None	-7.26E+05	5.00E+05	-1.4519086	0.1522079	
		Genotype_WT	3.70E+05	3.60E+05	1.0290105	0.3079789	
		CD8_Status:Colitis	1.80E+06	7.19E+05	2.5012698	0.0153814	*
	VMAT2	CD8_Status_Replete	7.38E+04	2.28E+05	0.3232843	0.7477059	
		Colitis_None	3.15E+04	2.25E+05	0.140118	0.8890787	
		Genotype_WT	2.99E+05	1.62E+05	1.8501015	0.0696737	
		CD8_Status:Colitis	4.34E+05	3.23E+05	1.3439693	0.1844763	

B.

Females	Variable	term	estimate	std.error	t.value	p.value	p.value.sig
	TH	CD8_Status_Replete	-5.09E+05	5.81E+05	-0.8754674	0.3848109	
		Colitis_None	1.12E+06	5.52E+05	2.0272233	0.0470891	*
		Genotype_WT	1.14E+06	4.05E+05	2.8200508	0.0064981	*
		CD8_Status:Colitis	-6.79E+05	8.13E+05	-0.8349514	0.4070575	
	pTH_TH	CD8_Status_Replete	2.52E-01	4.63E-02	5.4468692	0.0000013	*
		Colitis_None	-4.49E-02	4.48E-02	-1.0026455	0.3205038	
		Genotype_WT	-1.27E-01	3.24E-02	-3.9232142	0.000249	*
		CD8_Status:Colitis	-2.16E-01	6.50E-02	-3.332602	0.0015583	*
	DAT	CD8_Status_Replete	-1.15E+06	5.08E+05	-2.2732411	0.0269391	*
		Colitis_None	-7.26E+05	5.00E+05	-1.4519086	0.1522079	
		Genotype_WT	3.70E+05	3.60E+05	1.0290105	0.3079789	
		CD8_Status:Colitis	1.80E+06	7.19E+05	2.5012698	0.0153814	*
	VMAT2	CD8_Status_Replete	7.38E+04	2.28E+05	0.3232843	0.7477059	
		Colitis_None	3.15E+04	2.25E+05	0.140118	0.8890787	
		Genotype_WT	2.99E+05	1.62E+05	1.8501015	0.0696737	
		CD8_Status:Colitis	4.34E+05	3.23E+05	1.3439693	0.1844763	
